# Assessing the relative performance of fast molecular dating methods for phylogenomic data

**DOI:** 10.1186/s12864-022-09030-5

**Published:** 2022-12-03

**Authors:** Fernanda P. Costa, Carlos G. Schrago, Beatriz Mello

**Affiliations:** Department of Genetics, Federal University of Rio de Janeiro, Rio de Janeiro, RJ 21941-617 Brazil

**Keywords:** Bayesian analysis, Confidence interval, Divergence times, RelTime, TreePL, BEAST, MCMCTree, PhyloBayes

## Abstract

Advances in genome sequencing techniques produced a significant growth of phylogenomic datasets. This massive amount of data represents a computational challenge for molecular dating with Bayesian approaches. Rapid molecular dating methods have been proposed over the last few decades to overcome these issues. However, a comparative evaluation of their relative performance on empirical data sets is lacking. We analyzed 23 empirical phylogenomic datasets to investigate the performance of two commonly employed fast dating methodologies: penalized likelihood (PL), implemented in treePL, and the relative rate framework (RRF), implemented in RelTime. They were compared to Bayesian analyses using the closest possible substitution models and calibration settings. We found that RRF was computationally faster and generally provided node age estimates statistically equivalent to Bayesian divergence times. PL time estimates consistently exhibited low levels of uncertainty. Overall, to approximate Bayesian approaches, RelTime is an efficient method with significantly lower computational demand, being more than 100 times faster than treePL. Thus, to alleviate the computational burden of Bayesian divergence time inference in the era of massive genomic data, molecular dating can be facilitated using the RRF, allowing evolutionary hypotheses to be tested more quickly and efficiently.

**Supplementary Information:**

The online version contains supplementary material available at 10.1186/s12864-022-09030-5.

## Introduction

Molecular dating is an essential component of contemporary evolutionary studies. The idea that substitutions accumulate in a time-correlated manner in molecular sequences has greatly impacted evolutionary biology since it was proposed in the 1960s [1–4]. Over the last decades, major breakthroughs in sequencing technologies have allowed the assembly of large molecular datasets to estimate divergence times between species [5–8]. Such massive datasets pose a computational burden to parameter-rich molecular dating methods that rely on Bayesian Markov chain Monte Carlo (MCMC) sampling, slowing the testing and proposition of evolutionary hypotheses [9–12]. Because of this, phylogenomic studies have frequently devised alternative strategies to compute biological timescales, including the use of reduced datasets [13–19] and the summarization of time estimates based on data partitioning schemes [20, 21].

Such limitations prompted the development of rapid methods to date lineage divergences as alternatives to the standard Bayesian molecular dating, hence accelerating evolutionary analysis in the big data era [22, 23]. Like Bayesian approaches, the new methods have their own assumptions, including those related to how substitution rates vary across the phylogenetic tree. Currently, the most frequently used rapid molecular dating approaches are penalized likelihood (PL) [24] and the relative rate framework (RRF) [12, 25]. They have been employed in several branches of the Tree of Life, from prokaryotes to plants and animals [26–33]. Notably, these methodologies are more environmentally friendly than highly parametric Bayesian analyses, as their associated carbon footprints are orders of magnitude smaller [35]. Because of this, they might play an important role in the growing environmental awareness of bioinformatics research, conforming with the green computing standards [34, 35].

Although both PL and RRF do not require rate constancy, they are fundamentally distinct. PL uses a penalty function to minimize rate changes between adjacent branches globally [24]. Therefore, it assumes autocorrelation of evolutionary rates, which has been suggested as pervasive across the tree of life [36, 37]. A key component of PL is the smoothing parameter (λ), which controls the global level of rate variation and is optimized by a cross-validation method. The lower the value, the greater the rate variation across the phylogeny. PL was first implemented in the r8s software [38], and was later refined to deal with large phylogenies [39, 40]. In turn, RRF minimizes the difference in evolutionary rates of ancestral and descendant lineages individually [12]. This eliminates the need for a global penalty function and still accommodates rate differences between sister lineages [23]. As a result, RRF does not require any additional analytical step, such as the cross-validation procedure, to select an optimal level of rate variation. It is also important to mention that although the rates estimated by RRF are autocorrelated, RRF deals with lineage rates instead of branch rates [12], the standard modeling of Bayesian autocorrelated methods [41]. RRF is implemented in the RelTime routine of the software MEGA [42].

As they are currently implemented, PL and RRF also differ in the treatment of calibration information. While PL requires calibration information to be hard-bounded by minimum and/or maximum values [38], RRF via RelTime allows for the use of calibration densities [43]. Additionally, the uncertainty associated with the estimates of node ages are dealt with distinctly. PL can be combined with a bootstrap approach to asses uncertainty [38, 44], whereas RelTime adopts an explicit analytical equation to calculate confidence intervals [43]. Both frameworks reduce computational requirements compared to Bayesian relaxed clock methods. Because the algorithms of PL and RRF are different, results may be different, and their relative performances compared to Bayesian approaches have not been evaluated yet with empirical datasets.

As PL and RRF have been increasingly used to estimate timescales over the last years, it is essential to carry out large-scale evaluation against the popular Bayesian framework. While previous studies investigated both fast dating methods separately [22, 25, 40, 45–48], a joint assessment of their performance with empirical data is lacking [49]. Moreover, treePL, which is the most popular implementation of PL for large phylogenies, was not extensively compared to any Bayesian method whatsoever, and there is little information on how they behave comparatively with real data. In this regard, the phylogenomic datasets that have been produced in the last years provide the ideal opportunity to investigate the relative performances of rapid and Bayesian methods.

## Material and methods

We collected empirical datasets from 23 phylogenomic studies to assess the relative performance of fast dating methods compared to Bayesian methods. Studies were selected based on the availability of Bayesian timetrees or the input files used to carry out Bayesian inference plus molecular sequence alignments deposited in public databases or as supplementary information. Data retrieved comprise DNA and amino acid sequences from diverse taxonomic groups with divergences as old as the Precambrian. The number of sequences ranged from tens to nearly a thousand, and alignment lengths from ~ 5 kb to > 4 Mb. Alignment lengths, data types, number of terminals, calibration information, methodology originally employed, and the labels used to refer to each study, are summarized in Table 1.

**Table 1:** Detailed information about the phylogenomic datasets analyzed

Data reference	Label	Biological group	Data type^a^	Site count	Taxa count	Calibration count	Software used	Substitution model^b^
Allio et al. (2020) [50]	Allio20	Arthropoda	AA	288,446	61	5	PhyloBayes	JTT + F + G_5_ + I
Anderson et al. (2017) [51]	Anderson17	Annelida	AA	16,541	39	3	PhyloBayes	JTT + G_5_ + I
Blaimer et al. (2018) [52]	Blaimer18	Arthropoda	N	33,874	155	7	BEAST	GTR + G_5_
Borowiec (2019) [53]	Borowiec19	Arthropoda	N	44,079	162	3	BEAST	GTR + G_4_
Chazot et al. (2019) [54]	Chazot19	Arthropoda	N	6260	994	22	BEAST	GTR + G_5_
Delsuc et al. (2018) [55]	Delsuc18	Chordata	AA	66,593	63	11	PhyloBayes	LG + G_5_^c^
Delsuc et al. (2019) [56]	Delsuc19	Chordata	N	15,157	40	4	PhyloBayes	GTR + G_4_
dos Reis et al. (2018) [57]	dosReis18	Chordata	N	61,132	372	17	MCMCTree	GTR + G_4_
Fang et al. (2018) [58]	Fang18	Chordata	N	8079	128	3	BEAST	GTR + G_5_
Feng et al. (2017) [59]	Feng17	Chordata	N	88,302	164	20	MCMCTree	GTR + G_5_
Hedin et al. (2019) [60]	Hedin19	Arthropoda	N	71,483	27	3	PhyloBayes	GTR + G_5_
Hughes et al. (2018) [61]	Hughes18	Chordata	N	10,203	305	31	MCMCTree	HKY + G_5_
Irisarri et al. (2017) [20]	Irisarri17	Chordata	AA	14,043	100	14	PhyloBayes	JTT + F + G_4_ + I
Johnson et al. (2018) [62]	Johnson18	Arthropoda	N	131,013	193	23	MCMCTree	GTR + G_5_
Kuntner et al. (2019) [63]	Kuntner19	Arthropoda	N	89,212	34	2	MCMCTree	HKY + G_5_
Pereira et al. (2017) [64]	Pereira17	Chordata	N	12,354	294	22	MCMCTree	GTR + G_5_
Pessoa-Filho et al. (2017) [65]	PessoaFilho17	Streptophyta	N	135,255	30	1	BEAST	GTR + G_4_
Peters et al. (2017) [66]	Peters17	Arthropoda	AA	75,904	174	14	MCMCTree	JTT
Peters et al. (2018) [67]	Peters18	Arthropoda	AA	1,469,006	48	3	MCMCTree	JTT
Ran et al. (2018) [68]	Ran18	Streptophyta	N	4,246,454	16	4	MCMCTree	GTR + G_5_
Sann et al. (2018) [69]	Sann18	Arthropoda	N	284,607	184	10	MCMCTree	GTR + G_4_
Wolfe et al. (2019) [19]	Wolfe19	Arthropoda	AA	5994	95	19	PhyloBayes	JTT + G_4_^c^
Yonezawa et al. (2017) [70]	Yonezawa17	Chordata	N	873,274	45	6	MCMCTree	GTR + G_8_

^a^*N* nucleotide, *AA* amino acid

^b^The model that was used for most partitions, if applicable. The number of discrete categories to approximate the Gamma distributions is shown

The original studies have employed a Bayesian relaxed clock methodology as implemented in BEAST, MCMCTree, or PhyloBayes, except for Kuntner et al. (2019), who estimated divergence times using the RRF. In this case, the Bayesian timescale was inferred for the first time. Whenever possible, timetrees were directly obtained from the original works. Otherwise, divergence times were estimated using the input files published. We tried to keep substitution models matching the original studies, but studies that used CAT models of amino acid substitution implemented in PhyloBayes [71] were subjected to model selection in MEGA X [42]. If the original study applied data partitioning with distinct substitution models, we chose the model used in most partitions.

### Fast divergence time inference

We used the same alignment and topology as originally employed by the authors to estimate absolute times in RelTime [12, 25] and treePL [40]. Temporal calibration information was also extracted from the studies and applied according to the specificities of each method. To standardize computation, all analyses were carried out on a machine with a 3.2 GHz 6-Core Intel® i7 processor and 64 GB 2667 MHz DDR4 RAM. All branch lengths (in substitutions per site) used by both methods were estimated in MEGA X. RelTime calculations were performed with the command line version of MEGA X, and the confidence intervals (CI) of divergence times were calculated analytically, as implemented by the method.

In treePL, the program was firstly run using the option ‘prime’ to select the best optimization parameters. Then, a cross-validation procedure was performed to optimize the smoothing parameter values for each dataset [24], totalling 10 optimization iterations and 10^17^ simulated annealing iterations. The ‘cvstart’ and ‘cvstop’ parameters were set to 10^17^ and 10^− 19^, respectively, resulting in 37 smoothing parameter values tested. All analyses were run with the ‘thorough’ option. Confidence intervals of time estimates were calculated from 100 bootstrap replicates summarized in TreeAnnotator [72].

Regarding calibration information, whenever the original studies employed uniform priors, the bounds of the uniform distributions were provided as minimum and maximum boundaries of node age in treePL, while in RelTime, they were set as lower and upper limits of a uniform distribution. When probability distributions other than the uniform were originally used, namely, the normal, lognormal, exponential and skew-t distributions, they were also used in RelTime, except for the skew-t distribution, which is currently unavailable in this software. It was thus approximated by a normal distribution using the sn [73] and fitdistrplus packages [74] in R [75]. As treePL implements only minimum and maximum values as calibrations, we derived minimum and maximum bounds based on the lower 2.5% and upper 97.5% quantiles, respectively, of the density distributions. For the skew-t distribution, we did the same procedure, but using the normal distribution approximated for RelTime.

Because treePL works with rooted trees, the outgroup was removed before running the analyses. In RelTime, the outgroup was provided only to root the ingroup, but no calibrations were placed within it, and it was later removed from the estimated timetrees.

For the Kuntner et al. (2019) dataset, we inferred a Bayesian timescale in MCMCTree [76, 77] using the same calibration information, employing the independent rates prior with the HKY + G(5) substitution model [78]. Markov chain Monte Carlo analysis was run twice to check for convergence, each chain was sampled every 100th cycle until ESS values to approximate the posterior were greater than 200.

### Evaluation of relative performance

To contrast RelTime and treePL estimates to those derived with Bayesian methods, we calculated a series of metrics. For Bayesian time estimates, either the means or the medians of the posterior distribution of divergence times were used, depending on which value was reported in the original study. For each dataset, we performed linear regressions of RelTime and treePL estimates against Bayesian estimates. The coefficient of determination (R^2^) and the slope (β) of the linear regression through the origin were used as summary statistics to assess the strength of the association between fast and Bayesian dating methods.

For each data set, the average difference between fast dating methods and Bayesian time estimates was normalized to become comparable across studies that focused on various depths of the Tree of Life. Given *n* divergence times in a data set, for each *i*^th^ node age (*t*), the average difference was calculated as follows.

$$\overline{D}=\left(\frac{1}{n}\sum_{i=1}^n\frac{\mid {t}_{i, FAST}-{t}_{i, BAYES}\mid }{t_{i, BAYES}}\right)\times 100\%$$

Additionally, the precision of divergence time estimates was also accessed. For Bayesian time estimates, measures of uncertainty were as reported in the original study, either the highest posterior densities (HPDs) or the credibility intervals (CrIs). Because confidence and credibility intervals are fundamentally different from a statistical standpoint, they were not compared directly. In practice, these metrics are generally regarded as the measures of uncertainty associated with the time estimate in empirical studies, and they are required for evolutionary hypothesis testing. Thus, we reported their values for each method. For convenience, RelTime CIs, treePL CIs and HPDs/CrIs from Bayesian analyses will be hereafter referred to simply metrics of uncertainty.

For each dataset, two values were computed based on uncertainty metrics: the coverage and the median uncertainty width of each method. Coverage is a measure analogous to the success rate, as it indicates the frequency that node age estimates from fast methods were included within the credibility interval of the original Bayesian analyses. This frequency was computed for each dataset. The median uncertainty width of a method for each dataset was calculated as follows. For each *i*^th^ node age estimate, the difference between the maximum (*t*_max_) and minimum (*t*_min_) limits of the uncertainty metric (*U*) was normalized by the estimated node age (*t*).

$$U\ {width}_i=\frac{t_{i,\mathit{\max}}-{t}_{i,\mathit{\min}}}{t_i}$$

Therefore, uncertainty widths of a data set were transformed as fractions of the estimated node ages, and their median value was calculated. Importantly, this measure was computed excluding nodes that presented node ages smaller than 10^− 10^. This was done to avoid division by values near zero.

We tested whether the number of terminals, the number of sites in the alignment, and the percentage of calibrated nodes (the number of calibrations divided by the number of tree nodes) impacted the association between the Bayesian estimates and those from both fast-dating methods. Linear models were inferred using 1) the absolute deviations of the slope of the regression lines from 1 or 2) the mean squared errors (MSEs) as response variables. Besides MSE, we also tested the *R*^2^ and the RMSE as measures of goodness of fit with identical results. The importance of each feature was assessed by the varImp function [79] of the caret R package [80].

## Results

**Fig. 1:**
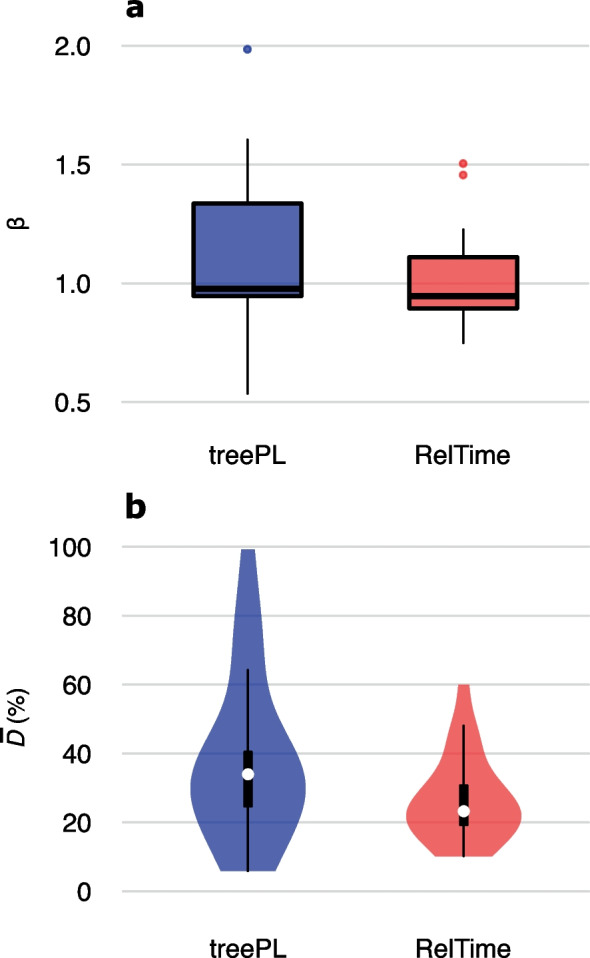
The performance of fast dating methodologies relative to the Bayesian methods for phylogenomic data. The slopes (β) of the linear regressions through the origin between rapid and Bayesian methods are shown in panel **a**. The mean normalized differences between RelTime/treePL and Bayesian node ages ($\overline{D}$) are shown in **b**

Fast methods produced time estimates highly correlated with Bayesian time estimates, regardless of the Bayesian method employed. All the recovered R^2^ values of the linear regression between fast methods and Bayesian node ages were ≥ 0.94, with most values higher than 0.98. The slope of the regression lines indicated a great correspondence between rapid methodologies and Bayesian node ages (Fig. 1a). The median slope values were 0.98 and 0.95 for treePL and RelTime, respectively. Nevertheless, the slopes of the regression lines between treePL and Bayesian time estimates presented a larger variance than when we compared RelTime to Bayesian node ages. For instance, in the Peters et al. dataset [67], the comparison of treePL and Bayesian time estimates returned a β = 1.99, indicating that node ages were generally 99% older than MCMCTree inferred times. For this same dataset, RelTime node ages led to a β = 1.46 when compared to Bayesian divergence times. For three other datasets, treePL estimates showed very high β values when compared to Bayesian estimates: PessoaFilho17 [65] (β_treePL_ = 1.57, β_RelTime_ = 1.15), Allio20 [50] (β_treePL_ = 1.58, β_RelTime_ = 1.09) and Peters17 [66] (β_treePL_ = 1.6, β_RelTime_ = 1.16). On the other hand, treePL produced much younger times for the dataset of Fang18 [58] (β_treePL_ = 0.54, β_RelTime_ = 0.75). The highest β recovered for RelTime was for the dataset of Ran18 [68] (β_RelTime_ = 1.5), which was very similar to the β recovered for treePL (β_treePL_ = 1.48). The lower β values produced by the node ages estimated by RelTime were for the datasets of Hedin19 [60] (β_treePL_ = 0.54, β_RelTime_ = 0.75) and Fang18 [58] (β_treePL_ = 0.78, β_RelTime_ = 0.75). Comparisons between time estimates using Bayesian and fast methods per dataset can be accessed through Supporting information 1.

The distribution of treePL $\overline{D}$ values was also wider than the distribution of RelTime (Fig. 1b). RelTime estimates were, on average, more similar to Bayesian time estimates, as the mean $\overline{D}$ was 26.5% for RelTime and 37.24% for treePL. When treePL was used to estimate divergence times, several datasets led to estimates that were, on average, more than 50% different from the Bayesian node ages. Conversely, RelTime molecular dates were, on average, more than 50% different than the Bayesian estimates for a single dataset (Ran18). For this dataset, both treePL and RelTime node ages were approximately 60% different from Bayesian times. For most datasets (70%), RelTime produced time estimates that were, on average, less than 30% different from the Bayesian ones, while treePL estimated node ages that were less than 30% distant from Bayesian times for only 39.13% of the datasets (Supporting information 2).

**Fig. 2:**
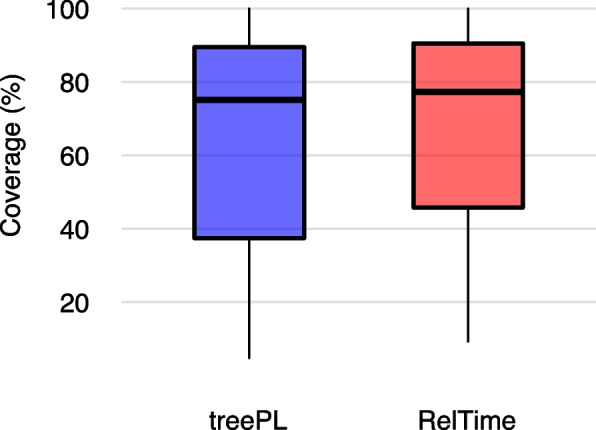
Frequency in which time estimates from treePL and RelTime were placed within the Bayesian credibility intervals as reported by the original studies (coverage)

Regarding the uncertainties of time estimates, treePL provided very narrow uncertainty intervals, with the distribution of the median interval widths across all datasets analyzed centered around 19.6%. This same value was centered around 64.3% for Bayesian and 102.3% for RelTime. For some of the datasets (52.17%), treePL uncertainty intervals eventually did not include the node ages estimated by the method itself. In these cases, up to 9% of the node ages did not fall within the intervals generated by treePL bootstrap approach. Regarding the frequency in which fast methods’ divergence times were included within the Bayesian credibility intervals, treePL and RelTime presented a similar performance. Mean coverage values for RelTime node ages were centered around 77.3%, while for treePL, it was placed around 75.1% (Fig. 2). The percentage of datasets that led to coverage values that included less than half of the estimated node ages of a phylogeny was 41% for treePL and 27% for RelTime. On the other hand, for 36 and 45% of the studies, time estimates were covered by the Bayesian credibility interval with a frequency of more than 80% when using treePL and RelTime, respectively.

For both fast-dating methods, deviation from the slope *β* =1 was significantly explained by the three features investigated (*p* < 0.001 and *R*^2^ = 0.59 for RelTime and *p* < 0.005 and *R*^2^ = 0.40 for treePL). The data feature with the highest importance in determining the deviation from a perfect fit to Bayesian estimates was the number of sites in the alignment (importance of 60% for RelTime and 37% for TrerePL). For explaining MSEs, the calibration density was the feature with the highest importance for RelTime (69%, *p* < 0.001 and *R*^2^ = 0.50), while treePL MSEs were not significantly predicted by any of the features analyzed (*p* > 0.05). For RelTime, increasing the density of calibrations resulted in more distinct time estimates from Bayesian analysis.

**Fig. 3:**
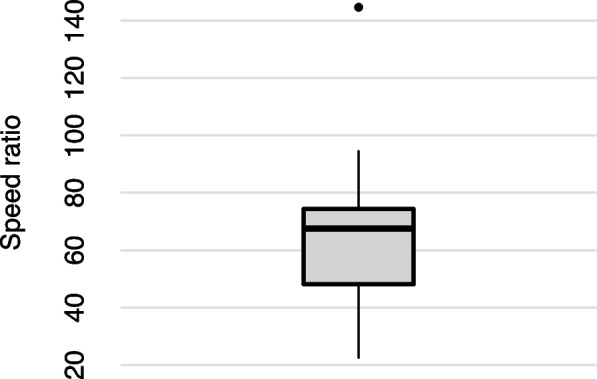
Computational speed ratio of RelTime to treePL for the phylogenomic datasets analyzed

Computational efficiency was very distinct between fast methods (Fig. 3). Average running times were 51.8 hours for treePL and 0.9 hours for RelTime. For most datasets, treePL took more than 24 hours to complete the calculations. In fact, RelTime usually took less than 2% of treePL running time, often more than 60 times faster than treePL (Fig. 3). Because confidence intervals are essential to retrieving uncertainty measures for divergence time estimates, treePL running times considered the estimation of branch lengths for the one hundred bootstrap replicates used to compute confidence intervals.

## Discussion

We provided the first comprehensive analysis of two of the most frequently used fast dating methodologies against Bayesian molecular dating, employing several empirical phylogenomic datasets from distinct biological groups, including up to hundreds of taxa. We measured differences in node age estimates, coverage of the Bayesian credibility intervals, and computational time efficiency. Our findings indicate that RRF, as implemented in RelTime, is a fast alternative to time-consuming molecular dating software. RelTime was much faster and generally provided time estimates closer to the Bayesian node ages than treePL. TreePL, which is considered a fast algorithm for performing molecular dating, required a significant computational time. This was due to the bootstrapping strategy used to compute confidence intervals of time estimates. As measurements of uncertainty are necessary to interpret biological scenarios derived from timetrees, their calculation entailed a running time that was comparable to Bayesian approaches, with some running times of more than one month.

Studies that have evaluated treePL performance against other approaches are scarce. The original work describing its implementation performed an evaluation using simulated and empirical data [40]. However, simulations did not include alignments, as the divergence times were directly inferred from the true tree, and the empirical datasets did not consist of several loci. Previous works employing both Bayesian approaches and treePL compared time estimates for specific taxa [81, 82], and their results are contrasting, with treePL leading either to older time estimates than BEAST in angiosperm evolution [82], or younger node ages than BEAST in a flowering plant family [81]. These works also reported contrasting results regarding the precision of treePL time estimates. In the present study, treePL confidence intervals were consistently narrow for all datasets analyzed. This result is expected because the bootstrap procedure leads to reduced parametric uncertainty as the number of sites increases, which is the case for phylogenomic data. Regarding time estimates, we found that treePL tended to produce older estimates than Bayesian analyses (Fig. 1a). This is in agreement with other works that have compared PL to Bayesian and non-Bayesian approaches [83–86].

It is already known that PL may provide overly ancient divergence time estimates when there is no calibration information to limit node ages near the root because of optimization issues [87]. The absence of efficient time constraints at deeper nodes was, in fact, common to all the analyses where older estimates were obtained (β > 1.1). For most of these datasets, treePL placed the age of the deep nodes precisely at or very close to the values provided as loose maxima. To test if the PL approach would present a better performance when outgroups and root/outgroup calibrations were kept in the analyses, we have conducted all treePL analyses using all ingroup and outgroup sequences and calibrations (when applicable). We did not find any significant performance improvement (Supporting information 3). Additionally, our findings corroborate Barba-Montoya et al. [49], which recovered a better performance for RelTime using simulated data. These authors found treePL to be more impacted by minor deviations from the molecular clock. While we have not quantified the clockness of the empirical datasets, this was probably one of the reasons for the more asymmetrical distributions of $\overline{D}$ values for treePL, while RelTime presented lower asymmetry (Supporting information 2).

Comparisons between time estimates retrieved by the RRF and Bayesian methods have been carried out in several empirical studies [12, 22, 25, 43, 45, 88–90]. Mello et al. (2017) and Tao et al. (2020) employed phylogenomic datasets and found that RelTime produced reliable time estimates compared to BEAST and MCMCTree. Here, we extended these findings to PhyloBayes software, which implements more sophisticated substitution models. Although MEGA does not provide the option to use the site-heterogeneous models implemented in PhyloBayes, times inferred employing the simpler models available in MEGA exhibited good correspondence to PhyloBayes estimates. The equivalence between timescales from simple and complex homogeneous substitution models was reported elsewhere [91]. We confirmed this finding and showed that it could be extended to site-heterogeneous substitution models.

If researchers need a faster alternative to Bayesian dating, our work demonstrated the good performance of RelTime’s RRF when compared to treePL. Besides providing node ages closer to Bayesian estimates, RelTime inferred ages were placed within Bayesian credibility intervals more frequently. Recently, using simulated data, Barba-Montoya et al. [49] also recovered a greater accuracy for RelTime when compared to other fast dating methods, particularly when autocorrelated rates were used. We showed that for empirical phylogenomic datasets, in which the true rate model is unknown, RelTime also performed better than treePL to approximate the standard Bayesian procedure. Additionally, on average, treePL produced rather precise estimates. The narrow confidence intervals of treePL estimates were also previously recovered using simulated data [49]. Simulations also have shown that RelTime confidence intervals exhibit equivalent or greater coverage probabilities than Bayesian approaches [43].

Besides having good statistical proprieties, we expect fast dating methods to reduce computational time significantly. We demonstrated that, on average, RelTime was 60 times faster than treePL. In the age of big data, such speed-up makes large-scale biological hypothesis testing feasible. Moreover, previous works based on simulations that accessed PL performance against Bayesian approaches and RelTime found that it performed worse than these methods under various scenarios of heterogeneous rates [25, 92]. These findings, together with our results that certified the speed of RelTime, demonstrate the usefulness of the RRF in obtaining biological timescales for large datasets.

The discrepancy between divergence time estimates from fast-dating and Bayesian methods was primarily influenced by the alignment length. Longer alignments resulted in larger differences between methods. This result is expected if methods rely on different modeling assumptions regarding parameters and evolutionary rate variation. Consequently, as the sample size approaches infinity, estimates become significantly different. For RelTime, calibration density significantly impacted the MSE of time estimates, implying that, besides alignment length, increasing the number of time constraints also makes the differences between methods more pronounced [49].

While previous work has advocated that RRF may not be suitable to infer divergence times for deep time datasets, leading to overly older time estimates [90], our analyses did not support this claim. Also, in contrast with a previous study [89], our results indicate that the strategy used by RelTime to calibrate timetrees [43] is as appropriate as the Bayesian calibration priors, yielding excellent correspondence between the timescales from both methods for most of the datasets (for ~ 78% of the datasets, β values deviated less than 0.2 from 1).

It is worth mentioning that larger differences between Bayesian analysis and RelTime may be retrieved at nodes connecting branches with lengths close to zero. Such lack of substitutions along branches causes RelTime to estimate more recent node ages. The fact that fast methods use branch lengths to estimate divergence times without relying on priors for node ages implies that when some branches have near zero substitutions, they underestimate times compared to Bayesian analysis. This occurs because divergence time priors assign lengths > 0 even when no substitutions are observed, as in the coalescent prior [93]. This may also affect treePL estimates, as observed for the dataset of Fang18 (Supporting information 2), although treePL may also assign non-zero time values to branches where the number of accumulated substitutions is effectively zero [40], leading to older inferred times than RelTime.

Our comparative analysis using a comprehensive empirical dataset has shown that fast dating methods are a viable alternative to time-consuming Bayesian methods to infer node ages for large-scale datasets. Additionally, we demonstrated that the RRF approach implemented in RelTime performed better, with lower demand in computational times. Thus, we emphasize the efficacy of the RRF in establishing molecular timescales with excellent correspondence to those inferred by Bayesian approaches. Timescales from different dating frameworks were impacted by alignment length, suggesting that their asymptotic properties are different. Furthermore, the quick estimation of confidence intervals of node ages allows for robust testing between several alternate evolutionary hypotheses, eliminating the computational burden brought forth by big data in biology.

## Supplementary Information

**Additional file 1: Supporting information 1.** Comparison of Bayesian and fast methods’ time estimates for each of the datasets analyzed. Each point represents an estimate of absolute time via Bayesian and treePL (blue) and RelTime (red) methods. Each graph contains the linear regression through the origin (dashed line), and the slope (β) and linear regression coefficient (R2) values. Calibrated nodes are represented by triangles. **Supporting information 2.** Kernel densities of the mean normalized differences between fast methods and Bayesian time estimates (Δt) for each of the datasets analyzed. For each node, it is computed as the difference between the treePL (blue) or RelTime (red) estimate and the Bayesian estimate divided by the Bayesian estimate. **Supporting information 3.** The performance of fast dating methodologies relative to the Bayesian methods for phylogenomic data. “RelTime” and “treePL (without calibrations)” are the same results presented in Fig. 1. “treePL (ape rooting)” and “treePL (Figtree rooting)” are the results recovered using the location of the root node placed by ape and Figtree, respectively. The slopes (β) of the linear regressions through the origin between rapid and Bayesian methods are shown in the left panels. The mean normalized differences between RelTime/treePL and Bayesian node ages (𝐷 “) are shown in the right panels.

## Data Availability

The datasets analyzed during the current study are available at the following link: https://github.com/biaumello/costa_et_al_2022.

## References

[1] Doolittle RF, Blomback B (1964). Amino-acid sequence investigations of Fibrinopeptides from various mammals: evolutionary implications. Nature.

[2] Margoliash E (1963). Primary structure and evolution of cytochrome C. Proc Natl Acad Sci U S A.

[3] Zuckerkandl E, Pauling L, Kasha M, Pullman B (1962). Molecular disease, evolution, and genic heterogeneity. Horizons in biochemistry.

[4] Zuckerkandl E, Pauling L. Evolutionary divergence and convergence in proteins. In: Evolving Genes and Proteins: Elsevier; 1965. p. 97–166. https://www.elsevier.com/books/evolving-genes-andproteins/bryson/978-1-4832-2734-4.

[5] Blair C, Bryson RW, Linkem CW, Lazcano D, Klicka J, McCormack JE (2019). Cryptic diversity in the Mexican highlands: thousands of UCE loci help illuminate phylogenetic relationships, species limits and divergence times of montane rattlesnakes (Viperidae: Crotalus ). Mol Ecol Resour.

[6] Givnish TJ, Zuluaga A, Spalink D, Soto Gomez M, Lam VKY, Saarela JM (2018). Monocot plastid phylogenomics, timeline, net rates of species diversification, the power of multi-gene analyses, and a functional model for the origin of monocots. Am J Bot.

[7] Tarver JE, dos Reis M, Mirarab S, Moran RJ, Parker S, O’Reilly JE (2016). The interrelationships of placental mammals and the limits of phylogenetic inference. Genome Biol Evol..

[8] Yang L, Su D, Chang X, Foster CSP, Sun L, Huang C-H (2020). Phylogenomic insights into deep phylogeny of angiosperms based on broad nuclear gene sampling. Plant Commun.

[9] Battistuzzi FU, Billing-Ross P, Paliwal A, Kumar S (2011). Fast and slow implementations of relaxed-clock methods show similar patterns of accuracy in estimating divergence times. Mol Biol Evol.

[10] Bromham L, Duchêne S, Hua X, Ritchie AM, Duchêne DA, Ho SYW (2018). Bayesian molecular dating: opening up the black box. Biol Rev Camb Philos Soc.

[11] Crosby RW, Williams TL (2017). Fast algorithms for computing phylogenetic divergence time. BMC Bioinformatics.

[12] Tamura K, Tao Q, Kumar S (2018). Theoretical Foundation of the RelTime method for estimating divergence times from variable evolutionary rates. Mol Biol Evol.

[13] Aardema ML, Stiassny MLJ, Alter SE (2020). Genomic analysis of the only blind cichlid reveals extensive inactivation in eye and pigment formation genes. Genome Biol Evol..

[14] Del Cortona A, Jackson CJ, Bucchini F, Van Bel M, D’hondt S, Škaloud P (2020). Neoproterozoic origin and multiple transitions to macroscopic growth in green seaweeds. Proc Natl Acad Sci U S A.

[15] Helmstetter AJ, Béthune K, Kamdem NG, Sonké B, Couvreur TLP (2020). Individualistic evolutionary responses of Central African rain forest plants to Pleistocene climatic fluctuations. Proc Natl Acad Sci U S A.

[16] Jarvis ED, Mirarab S, Aberer AJ, Li B, Houde P, Li C (2014). Whole-genome analyses resolve early branches in the tree of life of modern birds. Science..

[17] Percequillo AR, do Prado JR, Abreu EF, Dalapicolla J, Pavan AC, de Almeida Chiquito E (2021). Tempo and mode of evolution of oryzomyine rodents (Rodentia, Cricetidae, Sigmodontinae): a phylogenomic approach. Mol Phylogenet Evol.

[18] Smith SA, Brown JW, Walker JF (2018). So many genes, so little time: a practical approach to divergence-time estimation in the genomic era. PLoS One.

[19] Wolfe JM, Breinholt JW, Crandall KA, Lemmon AR, Lemmon EM, Timm LE (2019). A phylogenomic framework, evolutionary timeline and genomic resources for comparative studies of decapod crustaceans. Proc R Soc B Biol Sci.

[20] Irisarri I, Baurain D, Brinkmann H, Delsuc F, Sire J-Y, Kupfer A (2017). Phylotranscriptomic consolidation of the jawed vertebrate timetree. Nat Ecol Evol.

[21] Prum RO, Berv JS, Dornburg A, Field DJ, Townsend JP, Lemmon EM (2015). A comprehensive phylogeny of birds (Aves) using targeted next-generation DNA sequencing. Nature..

[22] Mello B, Tao Q, Tamura K, Kumar S (2017). Fast and accurate estimates of divergence times from big data. Mol Biol Evol.

[23] Tao Q, Tamura K, Kumar S, Ho SYW (2020). Efficient methods for dating evolutionary divergences. The molecular evolutionary clock.

[24] Sanderson MJ (2002). Estimating absolute rates of molecular evolution and divergence times: a penalized likelihood approach. Mol Biol Evol.

[25] Tamura K, Battistuzzi FU, Billing-Ross P, Murillo O, Filipski A, Kumar S (2012). Estimating divergence times in large molecular phylogenies. Proc Natl Acad Sci.

[26] Bond JE, Garrison NL, Hamilton CA, Godwin RL, Hedin M, Agnarsson I (2014). Phylogenomics resolves a spider backbone phylogeny and rejects a prevailing paradigm for orb web evolution. Curr Biol.

[27] Daane JM, Auvinet J, Stoebenau A, Yergeau D, Harris MP, Detrich HW (2020). Developmental constraint shaped genome evolution and erythrocyte loss in Antarctic fishes following paleoclimate change. PLoS Genet.

[28] Fernández-Mazuecos M, Vargas P, McCauley RA, Monjas D, Otero A, Chaves JA (2020). The radiation of Darwin’s Giant daisies in the Galápagos Islands. Curr Biol.

[29] Harvey MG, Bravo GA, Claramunt S, Cuervo AM, Derryberry GE, Battilana J (2020). The evolution of a tropical biodiversity hotspot. Science..

[30] Marin J, Battistuzzi FU, Brown AC, Hedges SB. The Timetree of prokaryotes: new insights into their evolution and speciation. Mol Biol Evol. 2016;34(2):437–446. 10.1093/molbev/msw245 27965376

[31] Qiao J, Zhang X, Chen B, Huang F, Xu K, Huang Q (2020). Comparison of the cytoplastic genomes by resequencing: insights into the genetic diversity and the phylogeny of the agriculturally important genus Brassica. BMC Genomics.

[32] Roxas BAP, Roxas JL, Claus-Walker R, Harishankar A, Mansoor A, Anwar F (2020). Phylogenomic analysis of Clostridioides difficile ribotype 106 strains reveals novel genetic islands and emergent phenotypes. Sci Rep.

[33] Shingate P, Ravi V, Prasad A, Tay B-H, Venkatesh B (2020). Chromosome-level genome assembly of the coastal horseshoe crab (Tachypleus gigas). Mol Ecol Resour.

[34] Grealey J, Lannelongue L, Saw W-Y, Marten J, Méric G, Ruiz-Carmona S (2022). The carbon footprint of bioinformatics. Mol Biol Evol.

[35] Kumar S (2022). Embracing green computing in molecular Phylogenetics. Mol Biol Evol.

[36] Lepage T, Bryant D, Philippe H, Lartillot N (2007). A general comparison of relaxed molecular clock models. Mol Biol Evol.

[37] Tao Q, Tamura K, U. Battistuzzi F, Kumar S. (2019). A machine learning method for detecting autocorrelation of evolutionary rates in large phylogenies. Mol Biol Evol.

[38] Sanderson MJ (2003). r8s: inferring absolute rates of molecular evolution and divergence times in the absence of a molecular clock. Bioinformatics..

[39] Paradis E (2013). Molecular dating of phylogenies by likelihood methods: a comparison of models and a new information criterion. Mol Phylogenet Evol.

[40] Smith SA, O’Meara BC (2012). treePL: divergence time estimation using penalized likelihood for large phylogenies. Bioinformatics..

[41] Kishino H, Thorne JL, Bruno WJ (2001). Performance of a divergence time estimation method under a probabilistic model of rate evolution. Mol Biol Evol.

[42] Kumar S, Stecher G, Li M, Knyaz C, Tamura K (2018). MEGA X: molecular evolutionary genetics analysis across computing platforms. Mol Biol Evol.

[43] Tao Q, Tamura K, Mello B, Kumar S (2020). Reliable confidence intervals for RelTime estimates of evolutionary divergence times. Mol Biol Evol.

[44] Paradis E, Claramunt S, Brown J, Schliep K. Confidence intervals in molecular dating by maximum likelihood. Mol Phylogenet Evol. 2022;178. 10.1016/j.ympev.2022.107652 36306994

[45] Battistuzzi FU, Tao Q, Jones L, Tamura K, Kumar S (2018). RelTime relaxes the strict molecular clock throughout the phylogeny. Genome Biol Evol..

[46] Chernikova D, Motamedi S, Csürös M, Koonin EV, Rogozin IB (2011). A late origin of the extant eukaryotic diversity: divergence time estimates using rare genomic changes. Biol Direct.

[47] Filipski A, Murillo O, Freydenzon A, Tamura K, Kumar S (2014). Prospects for building large Timetrees using molecular data with incomplete gene coverage among species. Mol Biol Evol.

[48] Gunter NL, Weir TA, Slipinksi A, Bocak L, Cameron SL (2016). If dung beetles (Scarabaeidae: Scarabaeinae) arose in association with dinosaurs, did they also suffer a mass co-extinction at the K-Pg boundary?. PLoS One.

[49] Barba-Montoya J, Tao Q, Kumar S (2021). Assessing rapid relaxed-clock methods for Phylogenomic dating. Genome Biol Evol..

[50] Allio R, Scornavacca C, Nabholz B, Clamens A-L, Sperling FA, Condamine FL (2020). Whole genome shotgun Phylogenomics resolves the pattern and timing of swallowtail butterfly evolution. Syst Biol.

[51] Anderson FE, Williams BW, Horn KM, Erséus C, Halanych KM, Santos SR (2017). Phylogenomic analyses of Crassiclitellata support major northern and southern hemisphere clades and a Pangaean origin for earthworms. BMC Evol Biol.

[52] Blaimer BB, Ward PS, Schultz TR, Fisher BL, Brady SG. Paleotropical diversification dominates the evolution of the Hyperdiverse ant tribe Crematogastrini (Hymenoptera: Formicidae). Insect Syst Divers. 2018;2(5):3; 1–14.

[53] Borowiec ML (2019). Convergent evolution of the Army ant syndrome and congruence in big-data Phylogenetics. Syst Biol.

[54] Chazot N, Wahlberg N, Freitas AVL, Mitter C, Labandeira C, Sohn J-C (2019). Priors and posteriors in Bayesian timing of divergence analyses: the age of butterflies revisited. Syst Biol.

[55] Delsuc F, Philippe H, Tsagkogeorga G, Simion P, Tilak M-K, Turon X (2018). A phylogenomic framework and timescale for comparative studies of tunicates. BMC Biol.

[56] Delsuc F, Kuch M, Gibb GC, Karpinski E, Hackenberger D, Szpak P (2019). Ancient Mitogenomes reveal the evolutionary history and biogeography of sloths. Curr Biol.

[57] dos Reis M, Gunnell GF, Barba-Montoya J, Wilkins A, Yang Z, Yoder AD (2018). Using Phylogenomic data to explore the effects of relaxed clocks and calibration strategies on divergence time estimation: Primates as a test case. Syst Biol.

[58] Fang B, Merilä J, Ribeiro F, Alexandre CM, Momigliano P (2018). Worldwide phylogeny of three-spined sticklebacks. Mol Phylogenet Evol.

[59] Feng Y-J, Blackburn DC, Liang D, Hillis DM, Wake DB, Cannatella DC (2017). Phylogenomics reveals rapid, simultaneous diversification of three major clades of Gondwanan frogs at the Cretaceous–Paleogene boundary. Proc Natl Acad Sci.

[60] Hedin M, Derkarabetian S, Alfaro A, Ramírez MJ, Bond JE (2019). Phylogenomic analysis and revised classification of atypoid mygalomorph spiders (Araneae, Mygalomorphae), with notes on arachnid ultraconserved element loci. PeerJ..

[61] Hughes LC, Ortí G, Huang Y, Sun Y, Baldwin CC, Thompson AW (2018). Comprehensive phylogeny of ray-finned fishes (Actinopterygii) based on transcriptomic and genomic data. Proc Natl Acad Sci.

[62] Johnson KP, Dietrich CH, Friedrich F, Beutel RG, Wipfler B, Peters RS (2018). Phylogenomics and the evolution of hemipteroid insects. Proc Natl Acad Sci.

[63] Kuntner M, Hamilton CA, Cheng R-C, Gregorič M, Lupše N, Lokovšek T (2019). Golden Orbweavers ignore biological rules: Phylogenomic and comparative analyses unravel a complex evolution of sexual size dimorphism. Syst Biol.

[64] Pereira AG, Sterli J, Moreira FRR, Schrago CG (2017). Multilocus phylogeny and statistical biogeography clarify the evolutionary history of major lineages of turtles. Mol Phylogenet Evol.

[65] Pessoa-Filho M, Martins AM, Ferreira ME (2017). Molecular dating of phylogenetic divergence between Urochloa species based on complete chloroplast genomes. BMC Genomics.

[66] Peters RS, Krogmann L, Mayer C, Donath A, Gunkel S, Meusemann K (2017). Evolutionary history of the Hymenoptera. Curr Biol.

[67] Peters RS, Niehuis O, Gunkel S, Bläser M, Mayer C, Podsiadlowski L (2018). Transcriptome sequence-based phylogeny of chalcidoid wasps (Hymenoptera: Chalcidoidea) reveals a history of rapid radiations, convergence, and evolutionary success. Mol Phylogenet Evol.

[68] Ran J-H, Shen T-T, Wu H, Gong X, Wang X-Q (2018). Phylogeny and evolutionary history of Pinaceae updated by transcriptomic analysis. Mol Phylogenet Evol.

[69] Sann M, Niehuis O, Peters RS, Mayer C, Kozlov A, Podsiadlowski L (2018). Phylogenomic analysis of Apoidea sheds new light on the sister group of bees. BMC Evol Biol.

[70] Yonezawa T, Segawa T, Mori H, Campos PF, Hongoh Y, Endo H (2017). Phylogenomics and morphology of extinct Paleognaths reveal the origin and evolution of the ratites. Curr Biol.

[71] Lartillot N, Philippe H (2004). A Bayesian mixture model for across-site heterogeneities in the amino-acid replacement process. Mol Biol Evol.

[72] Bouckaert R, Heled J, Kühnert D, Vaughan T, Wu C-H, Xie D (2014). BEAST 2: a software platform for Bayesian evolutionary analysis. PLoS Comput Biol.

[73] Azzalini A (2021). The R package “sn”: The Skew-Normal and Related Distributions such as the Skew-t and the SUN.

[74] Delignette-Muller ML, Dutang C. fitdistrplus : an R package for fitting distributions. J Stat Softw. 2015;64(4):1–34.

[75] R Core Team (2020). R: A language and environment for statistical computing.

[76] dos Reis M, Yang Z (2011). Approximate likelihood calculation on a phylogeny for Bayesian estimation of divergence times. Mol Biol Evol.

[77] Yang Z (2007). PAML 4: phylogenetic analysis by maximum likelihood. Mol Biol Evol.

[78] Hasegawa M, Kishino H, Yano T (1985). Dating of the human-ape splitting by a molecular clock of mitochondrial DNA. J Mol Evol.

[79] Gevrey M, Dimopoulos I, Lek S (2003). Review and comparison of methods to study the contribution of variables in artificial neural network models. Ecol Model.

[80] Kuhn M. Building predictive models in R using the caret package. J Stat Softw. 2008;28(5):1–26.

[81] Cai L, Xi Z, Peterson K, Rushworth C, Beaulieu J, Davis CC (2016). Phylogeny of Elatinaceae and the tropical Gondwanan origin of the Centroplacaceae (Malpighiaceae, Elatinaceae) clade. Plos one.

[82] Magallón S, Gómez-Acevedo S, Sánchez-Reyes LL, Hernández-Hernández T (2015). A metacalibrated time-tree documents the early rise of flowering plant phylogenetic diversity. New Phytol.

[83] Britton T, Anderson CL, Jacquet D, Lundqvist S, Bremer K (2007). Estimating divergence times in large phylogenetic trees. Syst Biol.

[84] Ericson PGP, Anderson CL, Britton T, Elzanowski A, Johansson US, Källersjö M (2006). Diversification of Neoaves: integration of molecular sequence data and fossils. Biol Lett.

[85] Mulcahy DG, Noonan BP, Moss T, Townsend TM, Reeder TW, Sites JW (2012). Estimating divergence dates and evaluating dating methods using phylogenomic and mitochondrial data in squamate reptiles. Mol Phylogenet Evol.

[86] Pérez-Losada M, Høeg JT, Crandall KA (2004). Unraveling the evolutionary radiation of the Thoracican barnacles using molecular and morphological evidence: a comparison of several divergence time estimation approaches. Syst Biol.

[87] Sanderson MJ, Thorne JL, Wikström N, Bremer K (2004). Molecular evidence on plant divergence times. Am J Bot.

[88] Battistuzzi FU, Billing-Ross P, Murillo O, Filipski A, Kumar S (2015). A protocol for diagnosing the effect of calibration priors on posterior time estimates: a case study for the Cambrian explosion of animal Phyla. Mol Biol Evol.

[89] Beavan AJS, Donoghue PCJ, Beaumont MA, Pisani D (2020). Performance of a priori and a posteriori calibration strategies in divergence time estimation. Genome Biol Evol..

[90] Lozano-Fernandez J, dos Reis M, Donoghue PCJ, Pisani D (2017). RelTime rates collapse to a strict clock when estimating the timeline of animal diversification. Genome Biol Evol.

[91] Tao Q, Barba-Montoya J, Huuki LA, Durnan MK, Kumar S (2020). Relative efficiencies of simple and complex substitution models in estimating divergence times in Phylogenomics. Mol Biol Evol.

[92] Ho SYW (2005). Accuracy of rate estimation using relaxed-clock models with a critical focus on the early metazoan radiation. Mol Biol Evol.

[93] Mello B, Tao Q, Barba-Montoya J, Kumar S (2021). Molecular dating for phylogenies containing a mix of populations and species by using Bayesian and RelTime approaches. Mol Ecol Resour.

