# Supplementary material for: Assessing the relative performance of fast molecular dating methods for phylogenomic data
Source: BMC Genomics. 2022 Dec 3;23:798. doi: 10.1186/s12864-022-09030-5 (PMC9719170; doi:10.1186/s12864-022-09030-5)

SUPPORTING INFORMATION

Accessing the relative performance of fast molecular dating methods for phylogenomic data

Fernanda P. Costa, Carlos G. Schrago and Beatriz Mello*

Department of Genetics, Federal University of Rio de Janeiro, RJ, Brazil

** Correspondence to:*

Beatriz Mello

Departamento de Genética, Instituto de Biologia, Universidade Federal do Rio de Janeiro

Rio de Janeiro, RJ 21941-617, Brazil

E-mail: bmello@biologia.ufrj.br

Supporting information 1: Comparison of Bayesian and fast methods' time estimates for each of the datasets analyzed. Each point represents an estimate of absolute time via Bayesian and treePL (blue) and RelTime (red) methods. Each graph contains the linear regression through the origin (dashed line), and the slope (β) and linear regression coefficient (R^2) values. Calibrated nodes are represented by triangles.

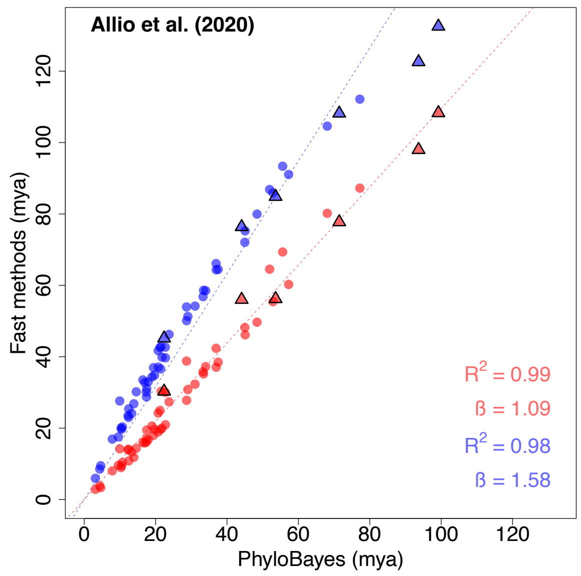

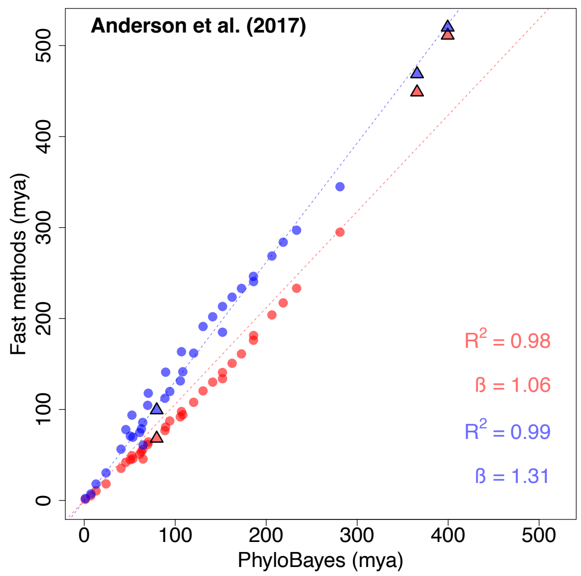

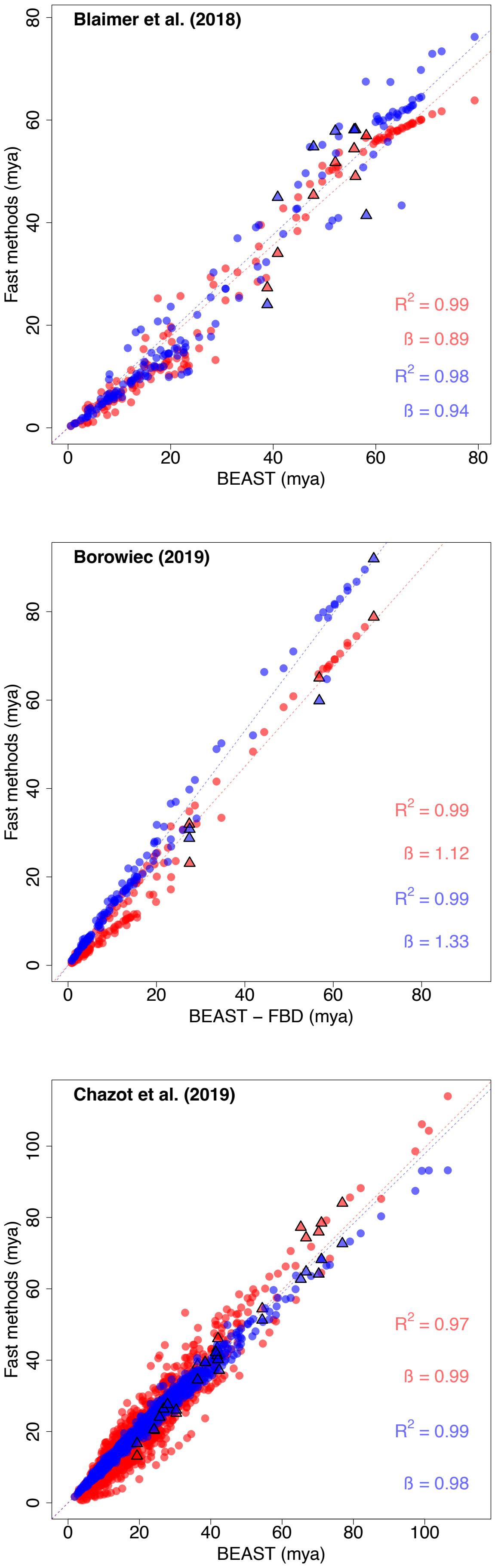

Blaimer et al. (2018)
Fast methods (mya)
BEAST (mya)
R2 = 0.99
ß = 0.89
R2 = 0.98
ß = 0.94
Borowiec (2019)
Fast methods (mya)
BEAST – FBD (mya)
R2 = 0.99
ß = 1.12
R2 = 0.99
ß = 1.33
Chazot et al. (2019)
Fast methods (mya)
BEAST (mya)
R2 = 0.97
ß = 0.99
R2 = 0.99
ß = 0.98

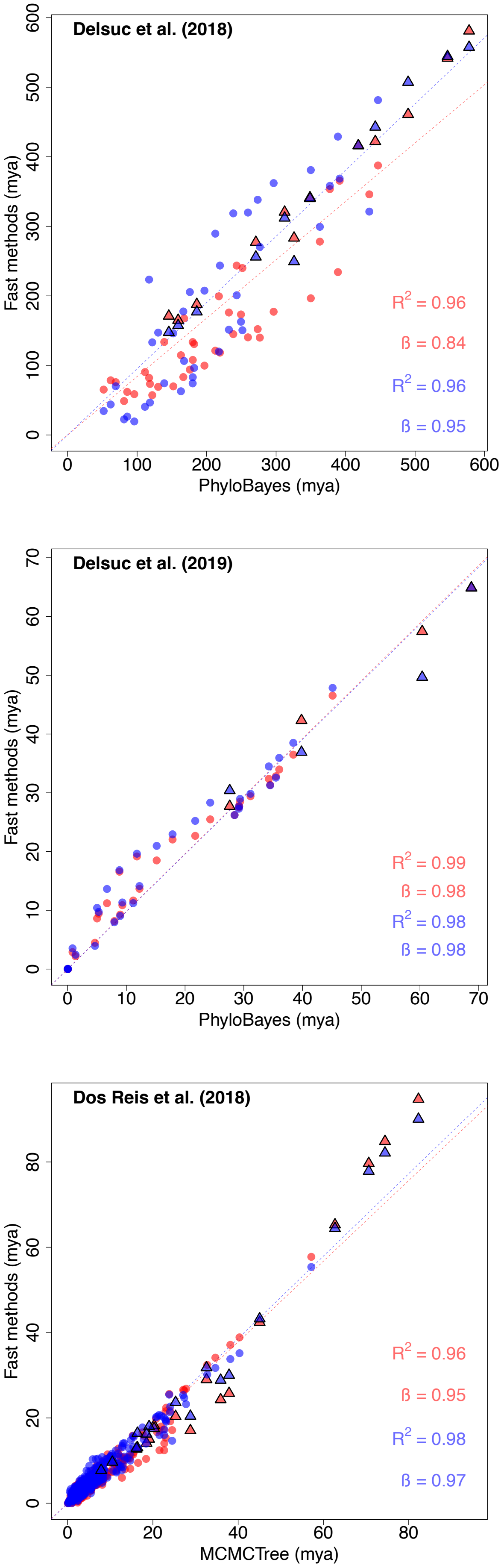

Delsuc et al. (2018)
Fast methods (mya)
PhyloBayes (mya)
R^2 = 0.96
ß = 0.84
R^2 = 0.96
ß = 0.95
Delsuc et al. (2019)
Fast methods (mya)
PhyloBayes (mya)
R^2 = 0.99
ß = 0.98
R^2 = 0.98
ß = 0.98
Dos Reis et al. (2018)
Fast methods (mya)
MCMCTree (mya)
R^2 = 0.96
ß = 0.95
R^2 = 0.98
ß = 0.97

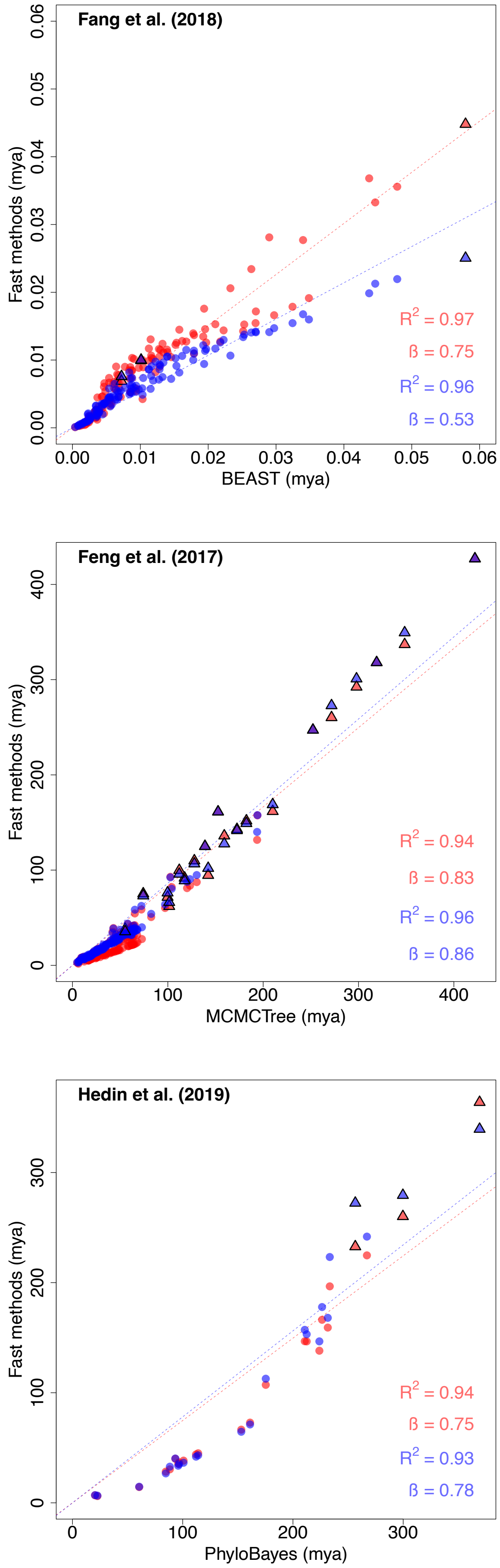

Fang et al. (2018)
Fast methods (mya)
BEAST (mya)
R2 = 0.97
ß = 0.75
R2 = 0.96
ß = 0.53
Feng et al. (2017)
Fast methods (mya)
MCMCTree (mya)
R2 = 0.94
ß = 0.83
R2 = 0.96
ß = 0.86
Hedin et al. (2019)
Fast methods (mya)
PhyloBayes (mya)
R2 = 0.94
ß = 0.75
R2 = 0.93
ß = 0.78

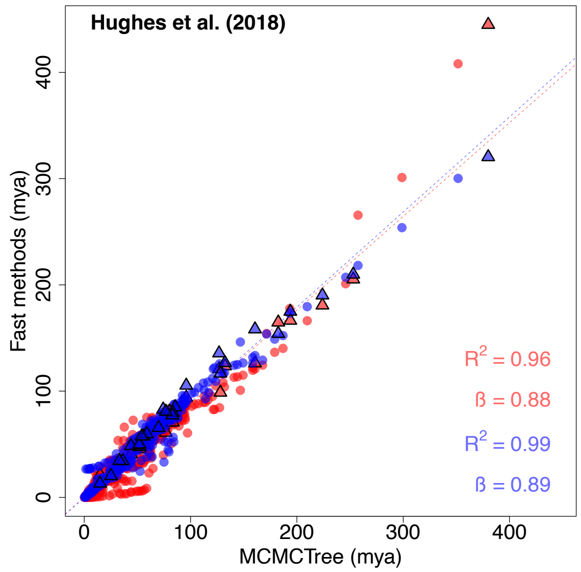
Hughes et al. (2018)
Fast methods (mya)
MCMCTree (mya)
R² = 0.96
ß = 0.88
R² = 0.99
ß = 0.89

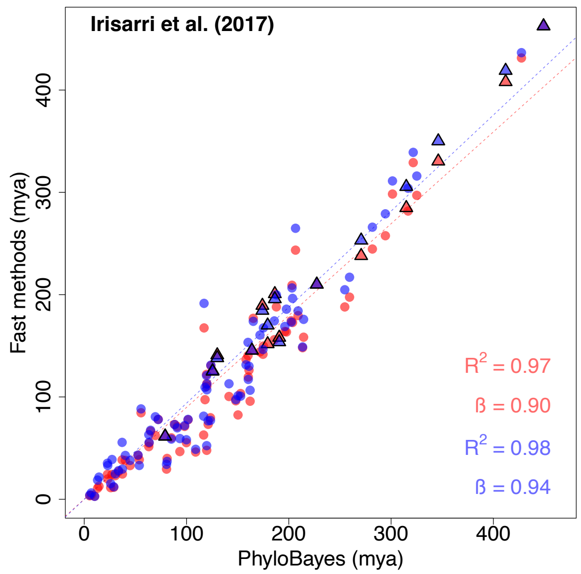
Irisarri et al. (2017)
Fast methods (mya)
PhyloBayes (mya)
R² = 0.97
ß = 0.90
R² = 0.98
ß = 0.94

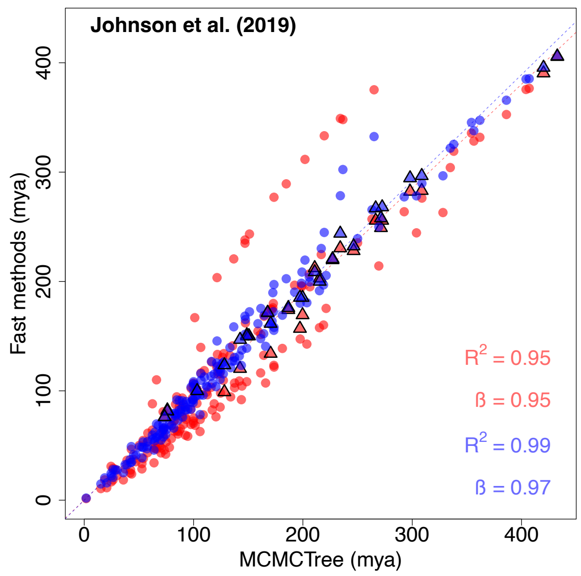
Johnson et al. (2019)
Fast methods (mya)
MCMCTree (mya)
R² = 0.95
ß = 0.95
R² = 0.99
ß = 0.97

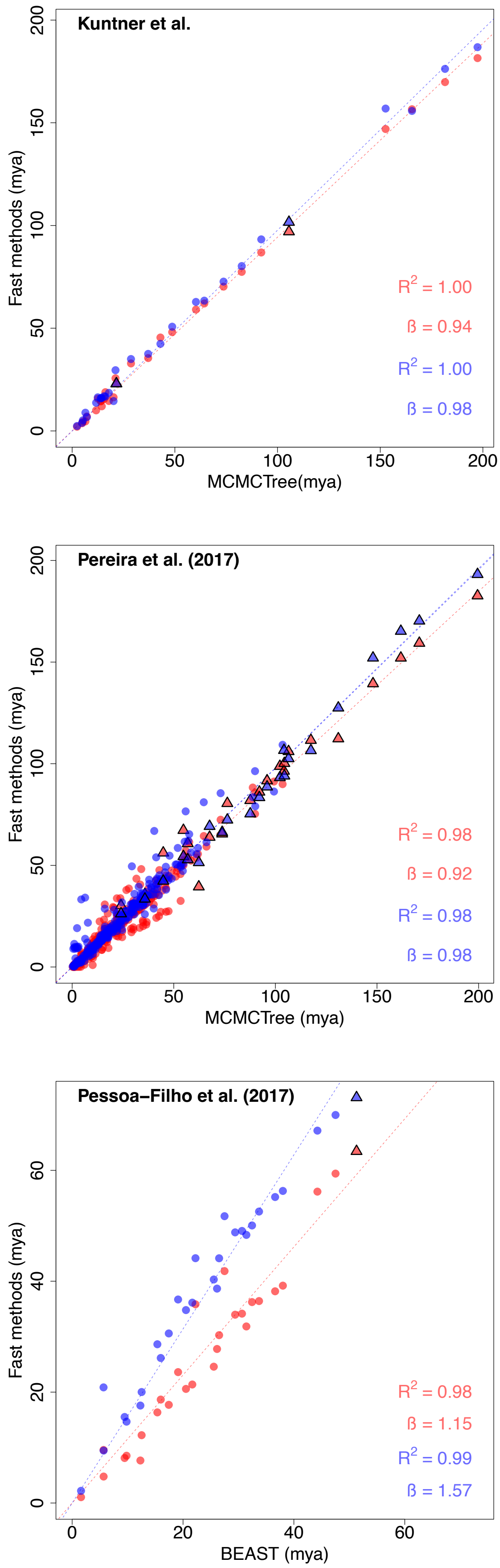
Kuntner et al.
Fast methods (mya)
MCMCTree(mya)
R2 = 1.00
ß = 0.94
R2 = 1.00
ß = 0.98
Pereira et al. (2017)
Fast methods (mya)
MCMCTree (mya)
R2 = 0.98
ß = 0.92
R2 = 0.98
ß = 0.98
Pessoa–Filho et al. (2017)
Fast methods (mya)
BEAST (mya)
R2 = 0.98
ß = 1.15
R2 = 0.99
ß = 1.57

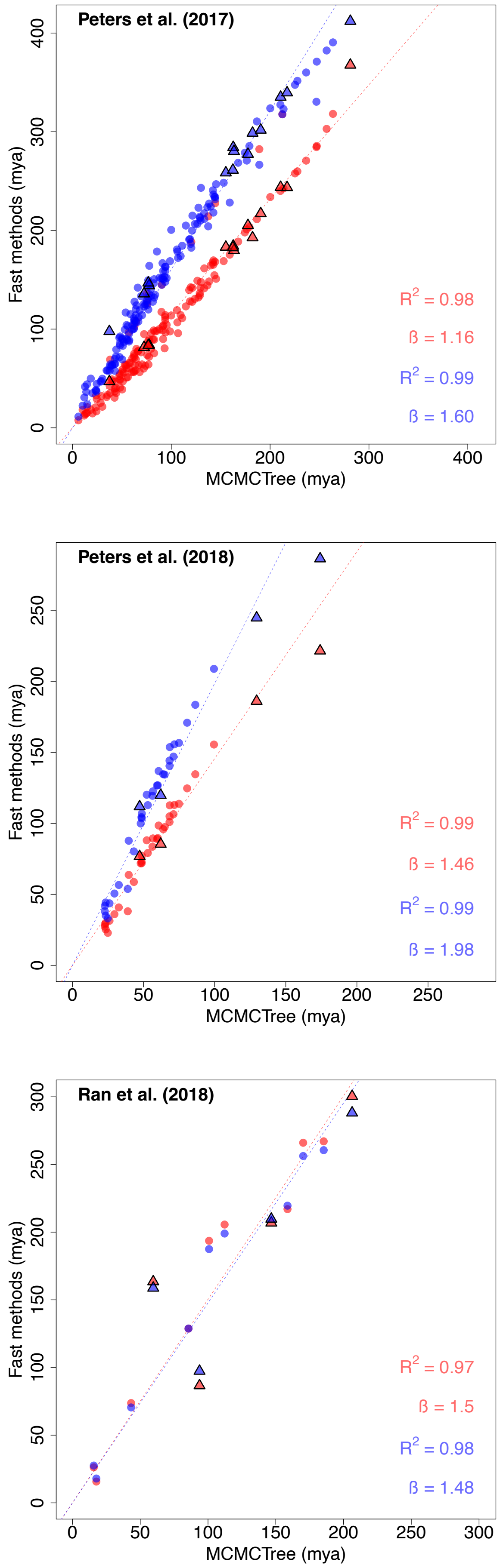

Peters et al. (2017)
Fast methods (mya)
MCMCTree (mya)
R2 = 0.98
ß = 1.16
R2 = 0.99
ß = 1.60
Peters et al. (2018)
Fast methods (mya)
MCMCTree (mya)
R2 = 0.99
ß = 1.46
R2 = 0.99
ß = 1.98
Ran et al. (2018)
Fast methods (mya)
MCMCTree (mya)
R2 = 0.97
ß = 1.5
R2 = 0.98
ß = 1.48

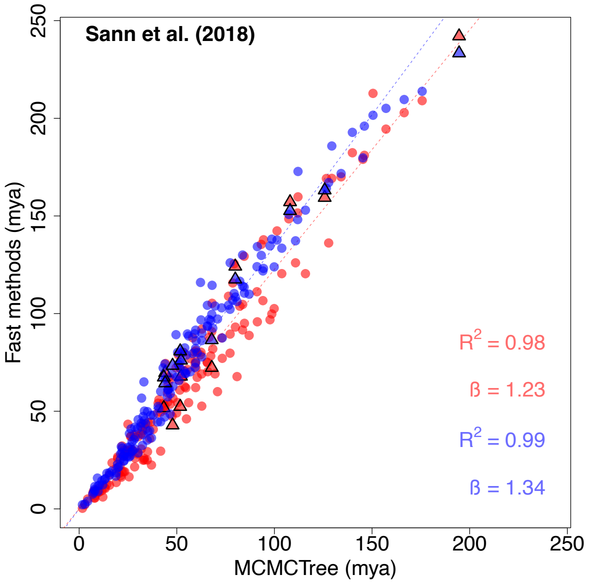
Sann et al. (2018)
Fast methods (mya)
MCMCTree (mya)
R2 = 0.98
ß = 1.23
R2 = 0.99
ß = 1.34

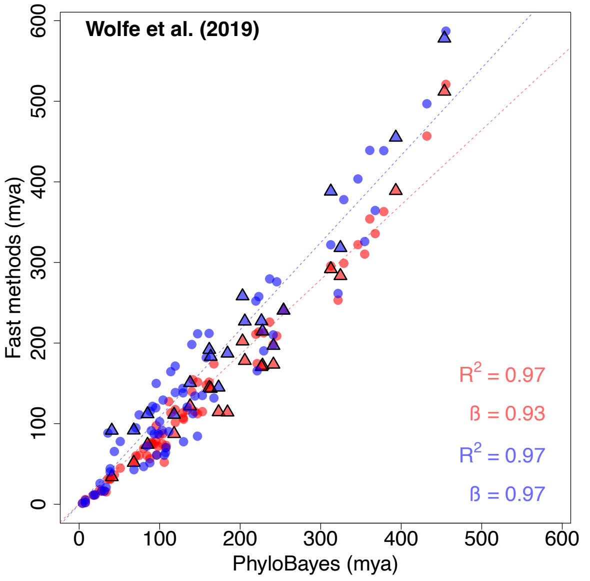
Wolfe et al. (2019)
Fast methods (mya)
PhyloBayes (mya)
R2 = 0.97
ß = 0.93
R2 = 0.97
ß = 0.97

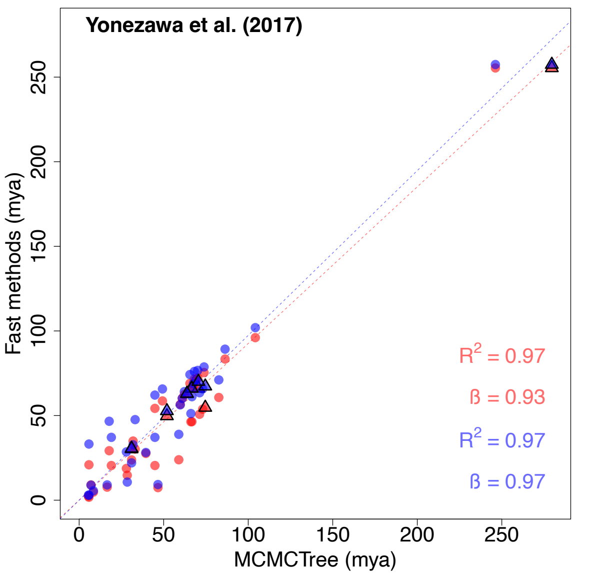
Yonezawa et al. (2017)
Fast methods (mya)
MCMCTree (mya)
R2 = 0.97
ß = 0.93
R2 = 0.97
ß = 0.97

Supporting information 2: Kernel densities of the mean normalized differences between fast methods and Bayesian time estimates (Δt) for each of the datasets analyzed. For each node, it is computed as the difference between the treePL (blue) or RelTime (red) estimate and the Bayesian estimate divided by the Bayesian estimate.

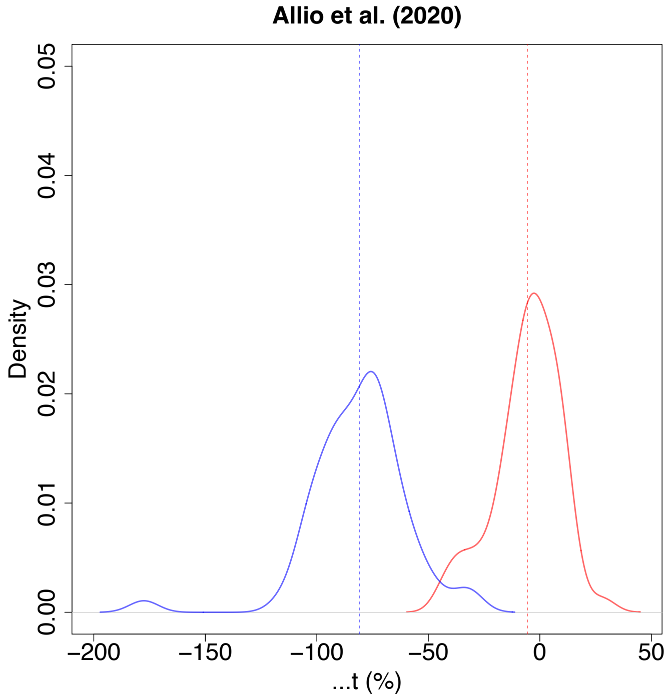

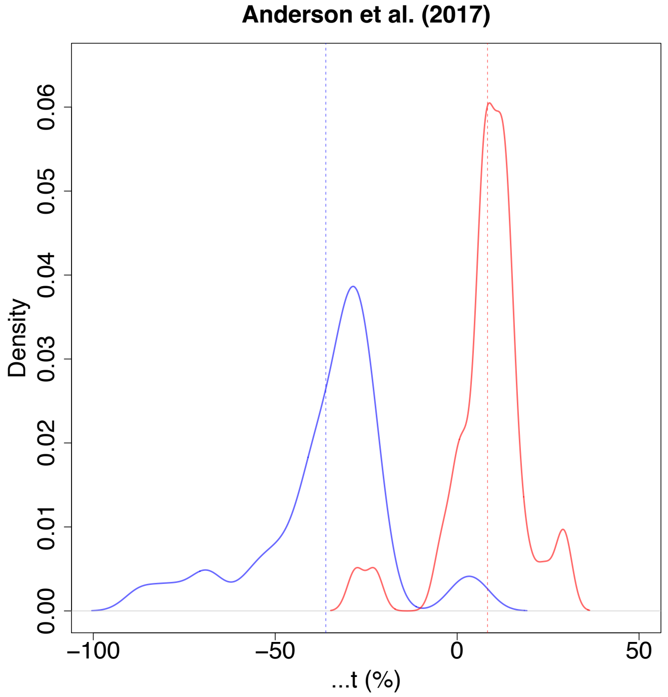

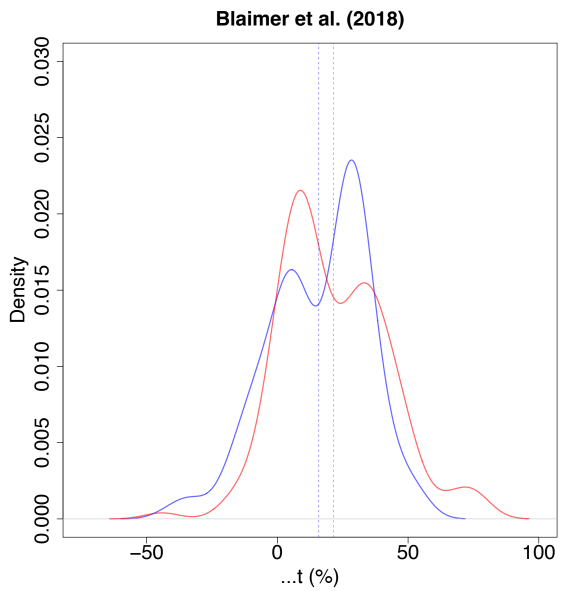
Blaimer et al. (2018)
Density
0.000
0.005
0.010
0.015
0.020
0.025
0.030
−50
0
50
100
…t (%)

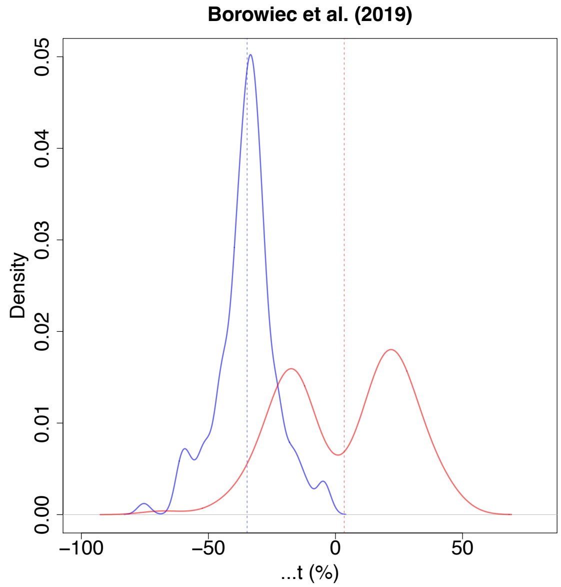
Borowiec et al. (2019)
Density
0.00
0.01
0.02
0.03
0.04
0.05
−100
−50
0
50
…t (%)

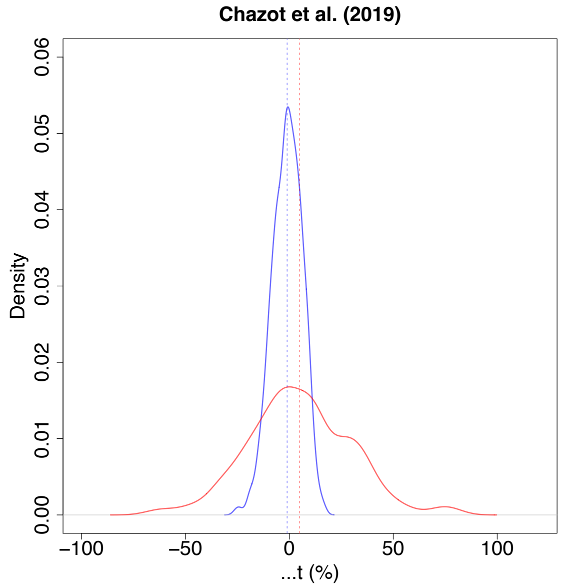
Chazot et al. (2019)
Density
0.00
0.01
0.02
0.03
0.04
0.05
0.06
−100
−50
0
50
100
…t (%)

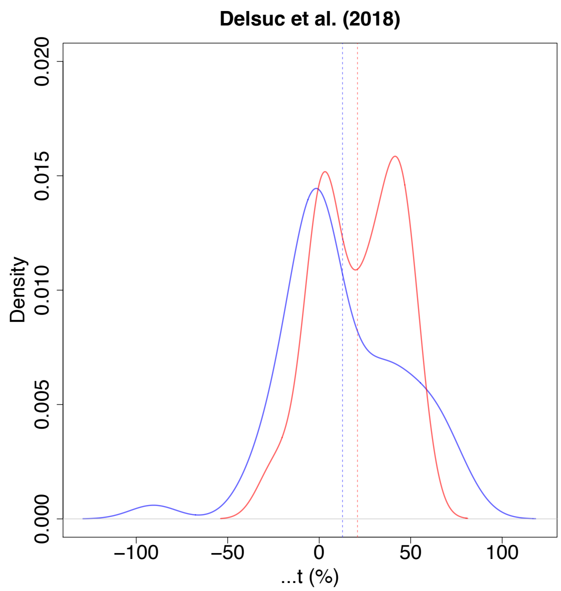
Delsuc et al. (2018)
Density
0.000
0.005
0.010
0.015
0.020
−100
−50
0
50
100
...t (%)

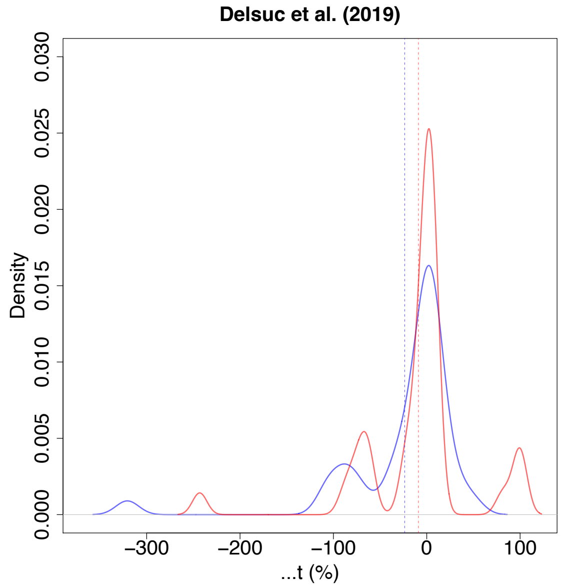
Delsuc et al. (2019)
Density
0.000
0.005
0.010
0.015
0.020
0.025
0.030
−300
−200
−100
0
100
...t (%)

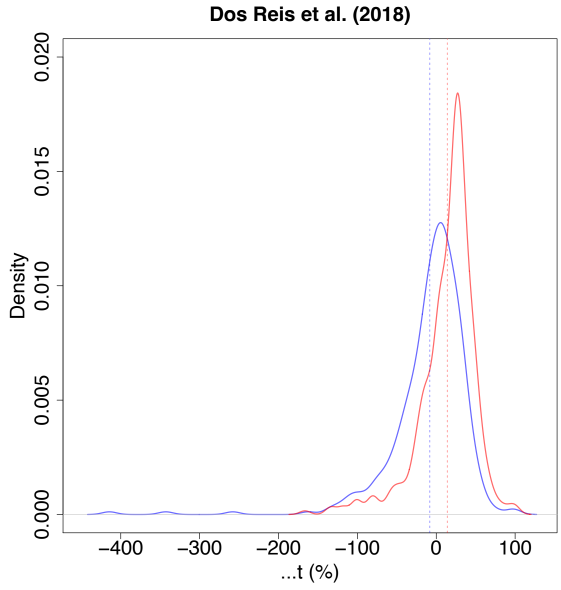
Dos Reis et al. (2018)
Density
0.000
0.005
0.010
0.015
0.020
−400
−300
−200
−100
0
100
...t (%)

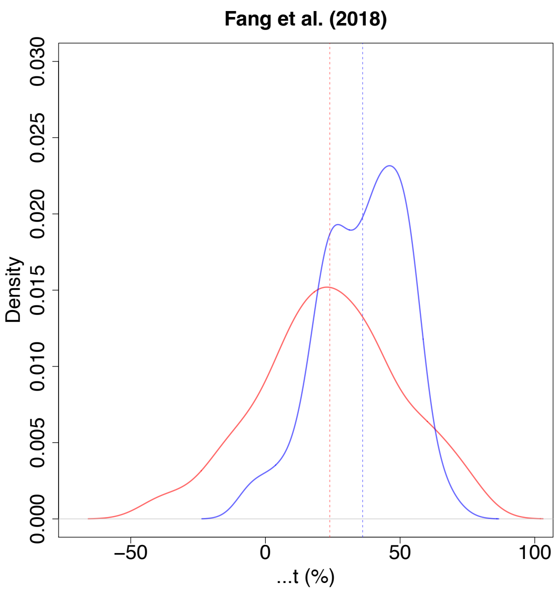
Fang et al. (2018)
Density
0.000
0.005
0.010
0.015
0.020
0.025
0.030
−50
0
50
100
...t (%)

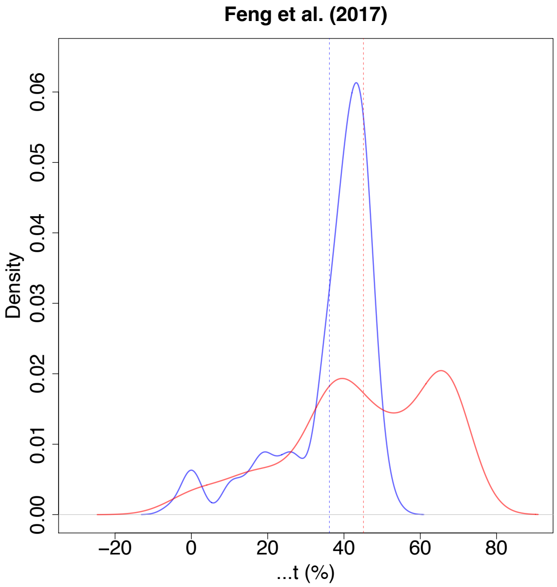
Feng et al. (2017)
Density
0.00
0.01
0.02
0.03
0.04
0.05
0.06
−20
0
20
40
60
80
...t (%)

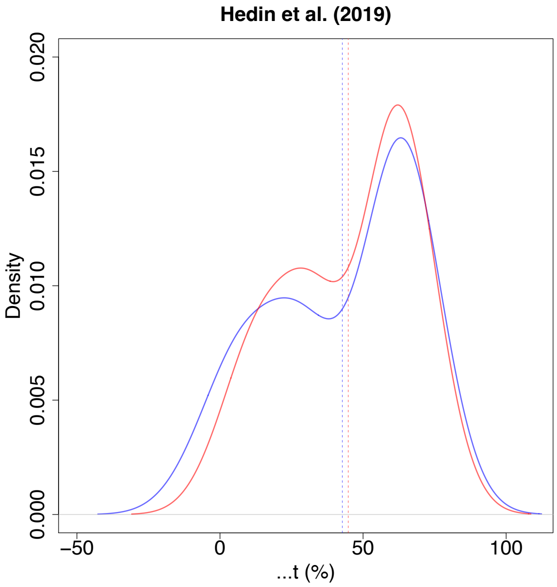
Hedin et al. (2019)
Density
0.000
0.005
0.010
0.015
0.020
−50
0
50
100
...t (%)

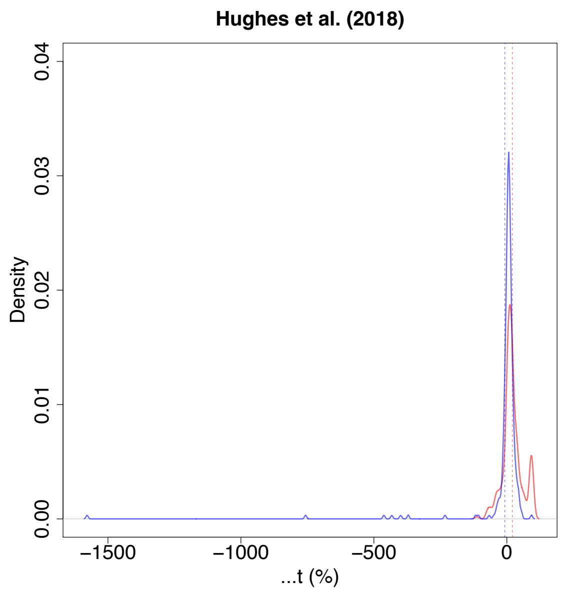
Hughes et al. (2018)
Density
0.00
0.01
0.02
0.03
0.04
−1500
−1000
−500
0
...t (%)

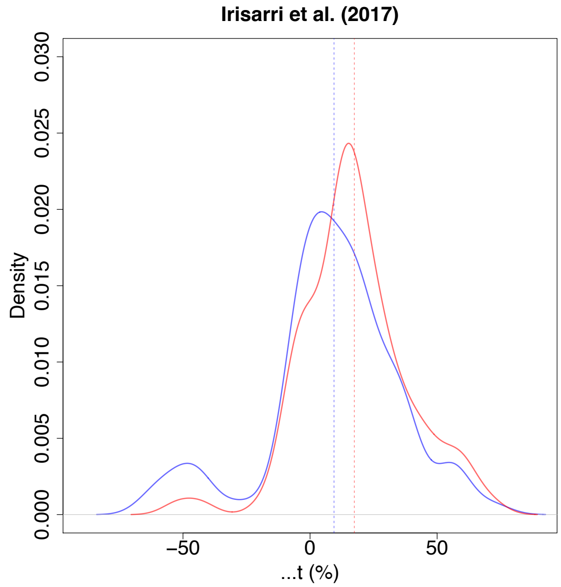
Irisarri et al. (2017)
Density
0.000
0.005
0.010
0.015
0.020
0.025
0.030
−50
0
50
...t (%)

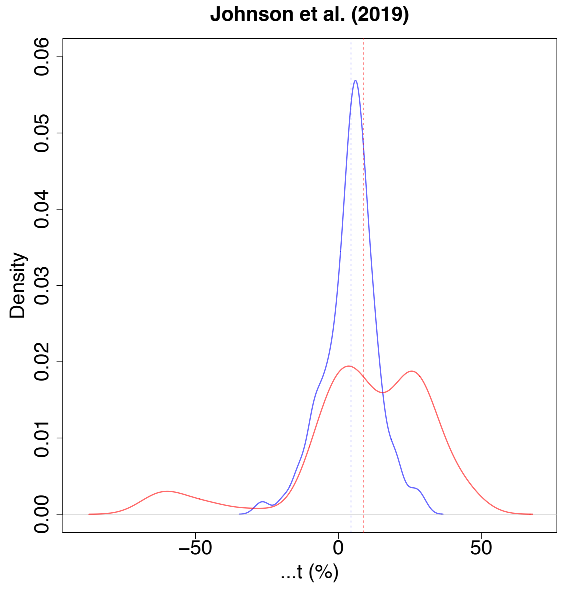
Johnson et al. (2019)
Density
0.00
0.01
0.02
0.03
0.04
0.05
0.06
−50
0
50
...t (%)

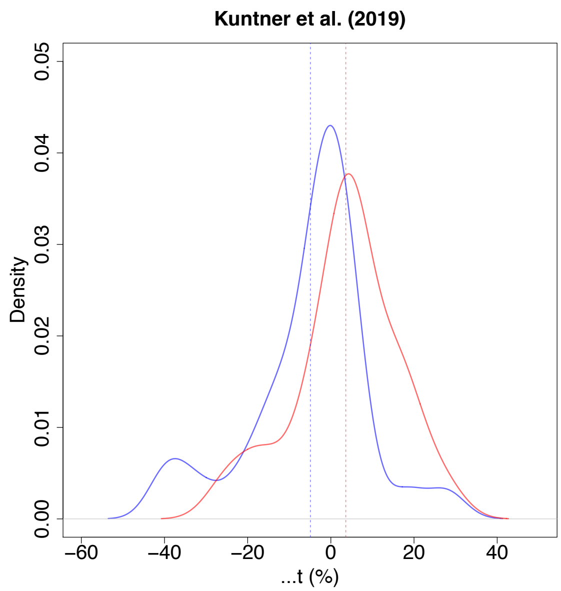
Kuntner et al. (2019)
Density
0.00
0.01
0.02
0.03
0.04
0.05
−60
−40
−20
0
20
40
...t (%)

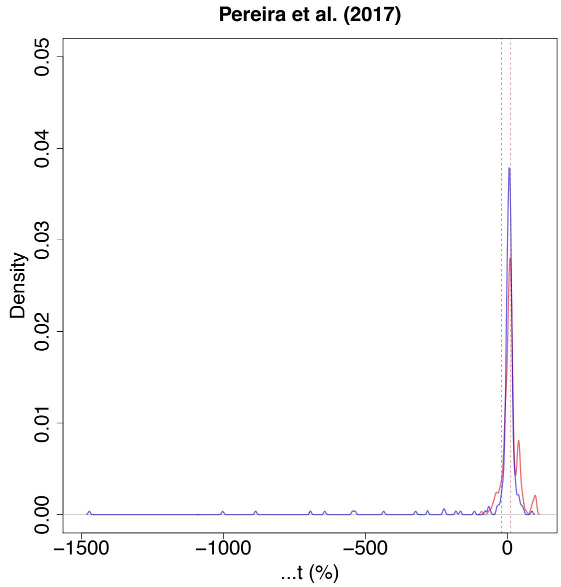
Pereira et al. (2017)
Density
0.00
0.01
0.02
0.03
0.04
0.05
−1500
−1000
−500
0
...t (%)

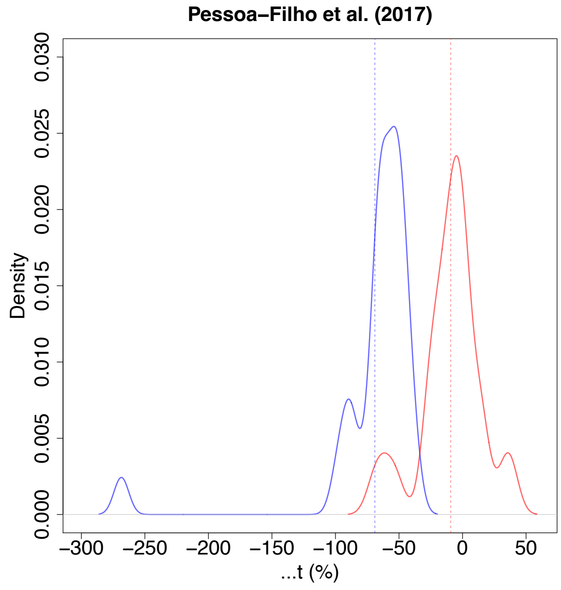
Pessoa−Filho et al. (2017)
Density
0.000
0.005
0.010
0.015
0.020
0.025
0.030
−300
−250
−200
−150
−100
−50
0
50
...t (%)

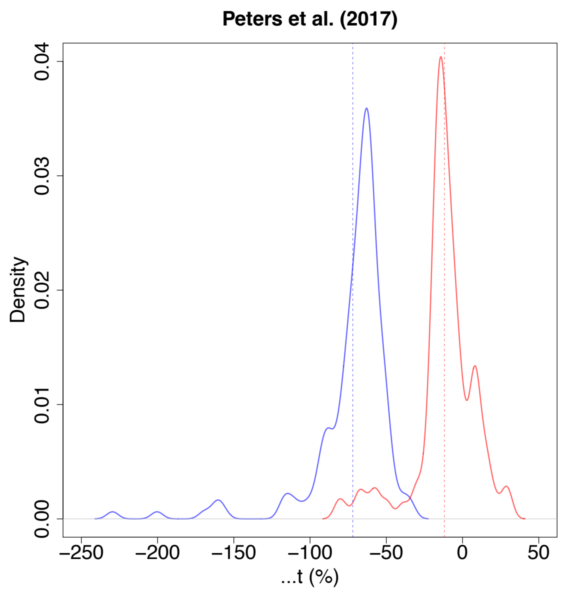
Peters et al. (2017)
Density
0.00
0.01
0.02
0.03
0.04
−250
−200
−150
−100
−50
0
50
...t (%)

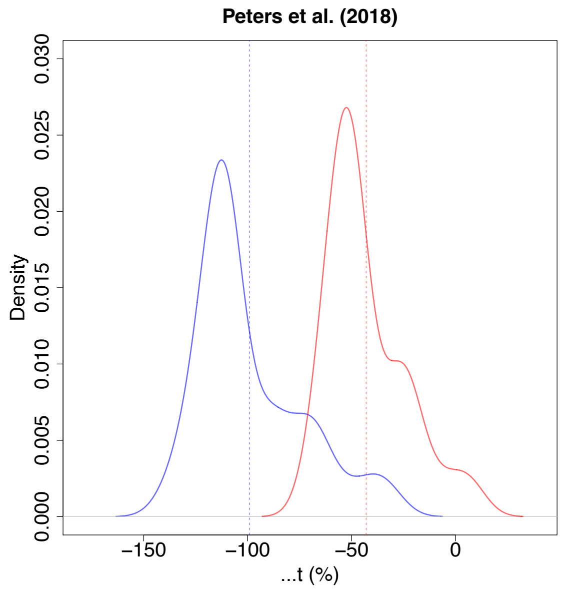
Peters et al. (2018)
Density
0.000
0.005
0.010
0.015
0.020
0.025
0.030
−150
−100
−50
0
...t (%)

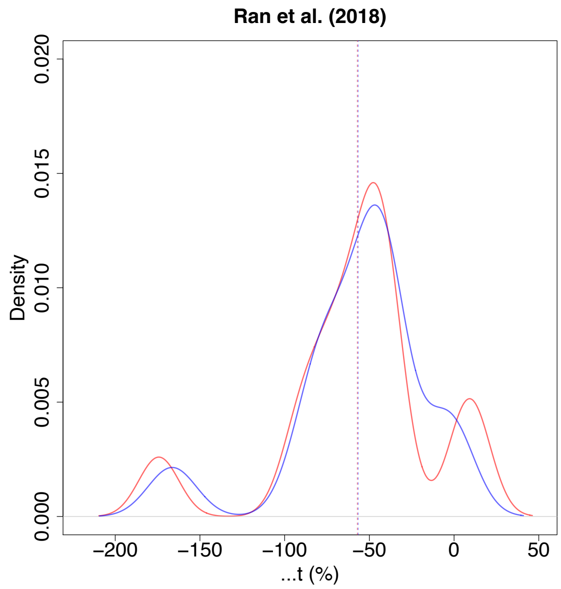
Ran et al. (2018)
Density
0.000
0.005
0.010
0.015
0.020
−200
−150
−100
−50
0
50
...t (%)

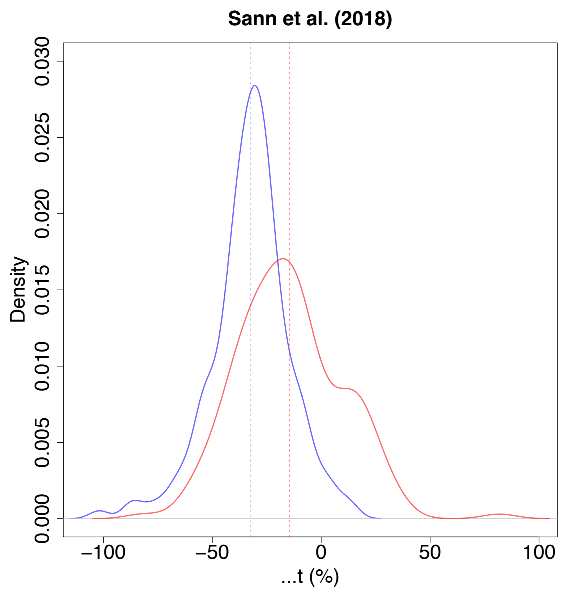

Sann et al. (2018)
Density
0.000
0.005
0.010
0.015
0.020
0.025
0.030
−100
−50
0
50
100
...t (%)

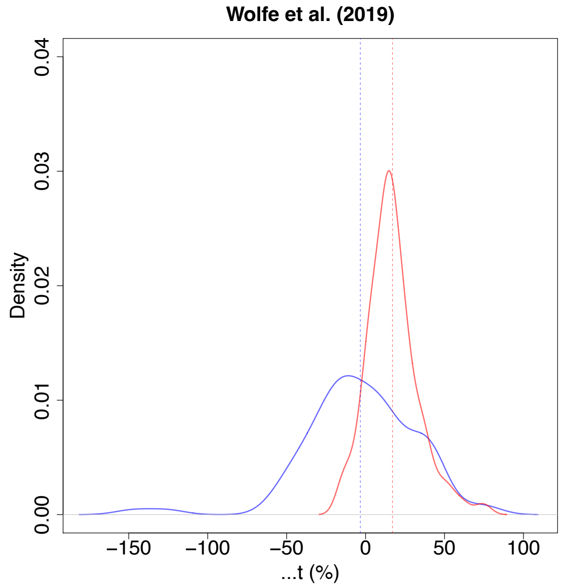

Wolfe et al. (2019)
Density
0.00
0.01
0.02
0.03
0.04
−150
−100
−50
0
50
100
...t (%)

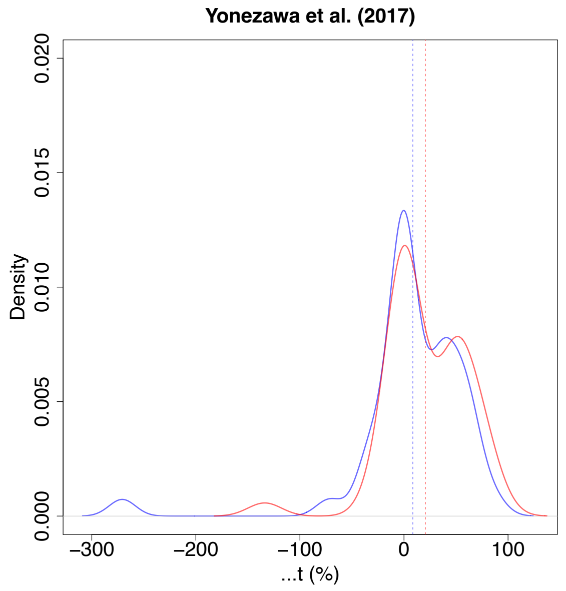

Yonezawa et al. (2017)
Density
0.000
0.005
0.010
0.015
0.020
−300
−200
−100
0
100
...t (%)

Supporting information 3: The performance of fast dating methodologies relative to the Bayesian methods for phylogenomic data. "RelTime" and "treePL (without calibrations)" are the same results presented in Figure 1. "treePL (ape rooting)" and "treePL (Figtree rooting)" are the results recovered using the location of the root node placed by ape and Figtree, respectively. The slopes (β) of the linear regressions through the origin between rapid and Bayesian methods are shown in the left panels. The mean normalized differences between RelTime/treePL and Bayesian node ages ($\overline{D}$) are shown in the right panels.

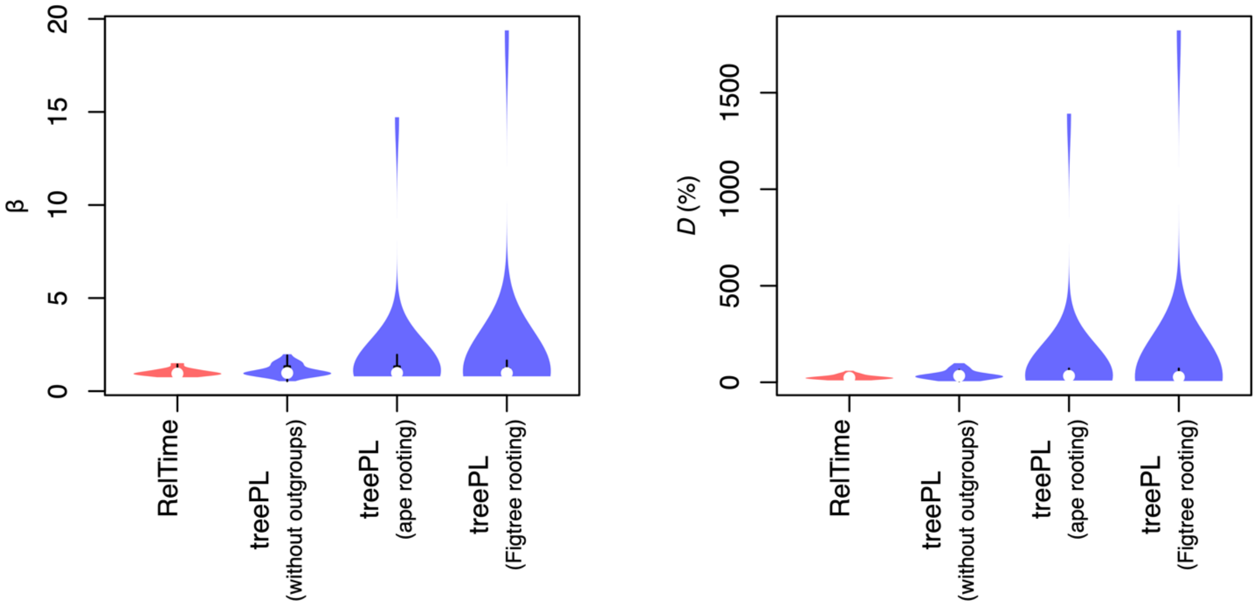

Supplement: Supplementary file 1 — Additional file 1: Supporting information 1. Comparison of Bayesian and fast methods’ time estimates for each of the datasets analyzed. Each point represents an estimate of absolute time via Bayesian and treePL (blue) and RelTime (red) methods. Each graph contains the linear regression through the origin (dashed line), and the slope (β) and linear regression coefficient (R2) values. Calibrated nodes are represented by triangles. Supporting information 2. Kernel densities of the mean normalized differences between fast methods and Bayesian time estimates (Δt) for each of the datasets analyzed. For each node, it is computed as the difference between the treePL (blue) or RelTime (red) estimate and the Bayesian estimate divided by the Bayesian estimate. Supporting information 3. The performance of fast dating methodologies relative to the Bayesian methods for phylogenomic data. “RelTime” and “treePL (without calibrations)” are the same results presented in Fig. 1. “treePL (ape rooting)” and “treePL (Figtree rooting)” are the results recovered using the location of the root node placed by ape and Figtree, respectively. The slopes (β) of the linear regressions through the origin between rapid and Bayesian methods are shown in the left panels. The mean normalized differences between RelTime/treePL and Bayesian node ages (𝐷 “) are shown in the right panels. [file 12864_2022_9030_MOESM1_ESM.pdf]
